# Emergency department attendance stratified by cause and frailty status: A national retrospective cohort study

**DOI:** 10.1111/ggi.70153

**Published:** 2025-08-28

**Authors:** Balamrit Singh Sokhal, Andrija Matetić, Joanne Protheroe, Toby Helliwell, Phyo K. Myint, Timir K. Paul, Christian D. Mallen, Mamas A. Mamas

**Affiliations:** ^1^ School of Medicine Keele University Keele UK; ^2^ Keele Cardiovascular Research Group, Centre for Prognosis Research Keele University Keele UK; ^3^ University Hospitals North Midlands Stoke‐on‐Trent UK; ^4^ Department of Cardiology University Hospital of Split Split Croatia; ^5^ Aberdeen Cardiovascular and Diabetes Centre, School of Medicine, Medical Sciences and Nutrition University of Aberdeen Aberdeen UK; ^6^ Institute of Applied Health Sciences, School of Medicine, Medical Sciences and Nutrition University of Aberdeen Aberdeen UK; ^7^ Ascension St. Thomas Hospital University of Tennessee Health Sciences Center at Nashville Nashville Tennessee USA

**Keywords:** emergency department, frailty, mortality, outcomes, risk factors

## Abstract

**Aim:**

The aim of this study was to determine whether the causes of emergency department (ED) attendance and clinical outcomes vary by frailty status.

**Methods:**

Using the Nationwide ED Sample, causes of attendance were stratified by Hospital Frailty Risk Score (HFRS). Logistic regression was used to determine adjusted odds ratios (aORs) and 95% confidence intervals (95% CIs) of ED and overall mortality.

**Results:**

A total of 155 497 048 ED attendances were included, of which 125 809 960 (80.9%) had a low HFRS (<5), 27 205 257 (17.5%) had an intermediate HFRS (5–15), and 2 481 831 (1.6%) had a high HFRS (>15). The most common cause of ED attendance in the high‐HFRS group was infectious diseases (43.0%), followed by cardiovascular diseases (CVD) (24.0%) and respiratory diseases (10.2%). For the low‐HFRS group, musculoskeletal disease was the most common cause (21.2%), followed by respiratory diseases (20.6%) and gastrointestinal diseases (18.5%). On adjusted analysis, high‐HFRS attendances had increased overall mortality (combined ED and in‐hospital) across most attendance causes, compared with their low‐risk counterparts (*P* < 0.001). High‐HFRS attendances with infectious diseases, CVD, and respiratory diseases had an increased risk of overall mortality, compared with their low‐risk counterparts (aOR 23.88, 95% CI 23.42–24.34 for the infectious disease cohort; aOR 2.58, 95% CI 2.55–2.61 for the CVD cohort; and aOR 36.90, 95% CI 36.18–37.62 for the respiratory disease cohort).

**Conclusions:**

Frailty is present in a significant proportion of ED attendances, with the cause varying by frailty status. Frailty is associated with decreased ED and increased overall mortality across most attendance causes. **Geriatr Gerontol Int 2025; 25: 1350–1358**.

## Graphical abstract

Intermediate and high frailty risk is present in a significant proportion of emergency department attendances, with the cause of attendance varying by frailty status. Frailty is associated with decreased emergency department mortality and increased in‐hospital mortality across most attendance causes.

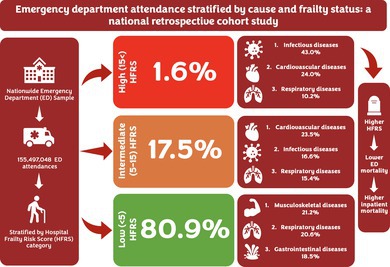

## Introduction

The population of older people is growing, and with it, the proportion of individuals living with frailty.^1^ Frailty is a clinical syndrome with impairment of multiple organ systems, leading to an increased vulnerability to stress, and is associated with an increased likelihood of adverse outcomes across a broad range of clinical conditions.^2^ Between 25% and 50% of people over 85 are estimated to be frail, with increased prevalence in females. In comparison to patients without frailty, frail patients have an increased length of hospital stay and incur higher total costs.^3^

There is little data on whether patient attributes differ by frailty status in the emergency department (ED) setting, including their prevalence, clinical characteristics, cause of attendance, and outcomes. The outcomes and disposition of patients in the ED vary: some presentations are resolved within the ED, including on‐site treatment and discharge; others are admitted for specialist inpatient hospital care; whilst others may result in death during the attendance.

Therefore, the aim of this study was to describe the prevalence of frailty in the ED setting, as well as to determine the association between frailty status and ED attendance cause, clinical characteristics, discharge disposition, and mortality of patients attending the ED.

## Methods

### Study dataset

The Nationwide ED Sample (NEDS) was developed by the Healthcare Cost and Utilization Project (HCUP) and is sponsored by the Agency for Healthcare Research and Quality (AHRQ).^4^ The NEDS provides estimates of all ED attendances in the United States, including 989 hospitals located in 40 states, amounting to approximately 145 million ED attendances. Patient demographics, comorbidities, and outcomes (including mortality) are all captured using International Classification of Diseases, Tenth Revision (ICD‐10) codes.^4^ The NEDS provides information about ED attendances only and cannot account for multiple presentations by the same person.

### The Hospital Frailty Risk Score

The Hospital Frailty Risk Score (HFRS) was developed by Gilbert *et al*. to assess the risk of adverse outcomes in older adult patients using electronic health record data.^5^ The HFRS was created using individually weighted ICD‐10 codes related to frailty, to stratify a cohort of older adult patients into low risk (HFRS <5), intermediate risk (HFRS 5–15), and high risk (HFRS >15) of frailty (Appendix S1).^5^ The score was validated using a local and national UK cohort.^5^ The original score was validated for over‐75 year‐olds; however, the score has since been used in various cohorts, including over‐18 year‐olds.^6^, ^7^, ^8^, ^9^, ^10^, ^11^

### Study population

Using the NEDS database, all adult ED attendances between 2016 and 2018 were detected and filtered by ICD‐10 chapters into 10 attendance causes of interest based on their primary discharge diagnosis. These groups were infectious diseases, neoplasms, hematological diseases, endocrine diseases, neurological diseases, cardiovascular diseases (CVD), respiratory diseases, gastrointestinal diseases, musculoskeletal diseases, and psychiatric diseases (Table S1). Attendance for other diagnostic reasons with individual prevalence <1.0% were deliberately excluded to enable a focus on the most important attendance causes. The sample was then stratified according to patients’ frailty status, measured by the HFRS as low, intermediate, and high risk, using ICD‐10 codes. All 40 diagnostic fields available to the NEDS were considered in this study.

The aims of this study were: (i) to determine the prevalence of frailty in the selected ED population; (ii) to determine the prevalence of different attendance causes across frailty groups; (iii) to compare patient characteristics across different attendance causes and frailty groups; (iv) to examine discharge disposition across different attendance causes and frailty groups; and (v) to determine mortality across different attendance causes and frailty groups. Discharge disposition included home, transfer to short‐term hospital, other transfer, home health care, discharge against medical advice, inpatient admission, and ED mortality. Mortality events in this study represent ED all‐cause mortality that occurred during the ED period, as well as overall all‐cause mortality that includes both the ED and in‐hospital period.

Cases were excluded owing to missing data for the following variables: age, sex, and overall mortality. These cases accounted for no more than 1.0% of the original dataset. As this is an observational study, it was appraised according to the Strengthening The Reporting of OBservational Studies in Epidemiology (STROBE) recommendations^12^ (Appendix S2).

### Statistical analysis

Continuous variables, including age, length of stay, and total charges, were summarised using median and interquartile ranges (IQRs) and compared using the Kruskal–Wallis test. Categorical variables were summarised as percentages (%) and compared using the chi‐squared (*X* ^2^) test. Results yielding low numbers of cases where percentages were less than 0.1% were expressed as <0.1%.

Binomial multivariable logistic regression was performed to determine the adjusted odds ratios (aORs) and 95% confidence intervals (95% CIs) for all‐cause mortality across frailty groups and different attendance causes. Variables that are intrinsically part of the HFRS were not included in the multivariable models to avoid multicollinearity. The logistic regression was adjusted for the following variables: age, sex, weekend admission, primary expected payer, median household income, hospital region and teaching status, previous acute myocardial infarction (AMI), dyslipidemia, smoking, coagulopathy, and chronic renal failure. These variables were selected based on their clinical relevance and prior literature. Statistical significance was set at the level of *P* < 0.05. All statistical analyses were weighted and performed using SPSS version 27 (IBM Corp, Armonk, NY).^4^

## Results

### Baseline characteristics

A total of 155 497 048 selected ED attendances with the attendance causes of interest were recorded between 2016 and 2018 (Fig. S1). Overall, 125 809 960 (80.9%) had a low HFRS, 27 205 257 (17.5%) had an intermediate HFRS, and 2 481 831 (1.6%) had a high HFRS (Table 1). When looking at the attendance causes, the most common cause of attendance was for respiratory diseases (*N* = 30 306 088 [19.5%]), followed by musculoskeletal diseases (*N* = 28 537 858 [18.4%]), gastrointestinal diseases (*N* = 27 035 858 [17.4%]), CVD (*N* = 20 498 940 [13.2%]), psychiatric diseases (*N* = 15 725 433 [10.1%]), infectious diseases (*N* = 11 201 562 [7.2%]), neurological diseases (*N* = 9 921 873 [6.4%]), endocrine diseases (*N* = 7 862 684 [5.1%]), hematological diseases (*N* = 2 296 711 [1.5%]) and neoplasms (*N* = 2 110 162 [1.4%]) (Table 2).

**Table 1:** Patient characteristics stratified by HFRS

Characteristics	Hospital Frailty Risk Score			*P*‐value
	Low <5 (80.9%)	Intermediate 5–15 (17.5%)	High >15 (1.6%)	
Number of weighted discharges	125 809 960	27 205 257	2 481 831	
Age (years), median (IQR)	57 (42, 71)	69 (57, 80)	77 (66, 85)	<0.001
Female sex, %	55.0	52.8	55.0	<0.001
Weekend admission, %	27.2	25.6	25.9	<0.001
Primary expected payer, %				<0.001
Medicare	26.9	64.9	79.9	
Medicaid	26.8	13.5	8.2	
Private insurance	28.0	15.3	8.9	
Self‐pay	14.3	4.0	1.5	
No charge	0.6	0.3	0.1	
Other	3.5	2.0	1.4	
Median household income (percentile), %				<0.001
0–25th	36.7	32.9	30.6	
26th–50th	27.6	26.4	25.1	
51st–75th	20.4	22.0	22.7	
76th–100th	15.4	18.7	21.6	
Comorbidities, %				
Dyslipidaemia	10.7	41.0	45.1	<0.001
Thrombocytopenia	0.7	6.5	9.7	<0.001
Smoking	15.3	14.1	7.6	<0.001
Previous AMI	2.0	8.2	7.2	<0.001
Previous PCI	1.6	7.4	5.6	<0.001
Previous CABG	1.3	6.6	5.7	<0.001
Anemias	3.7	28.4	39.1	<0.001
Valvular disease	0.9	7.5	9.2	<0.001
Peripheral vascular disorders	0.3	3.4	4.6	<0.001
Coagulopathy	1.1	8.5	12.8	<0.001
Diabetes mellitus	13.8	38.2	40.3	<0.001
Liver disease	1.3	6.2	5.6	<0.001
Chronic renal failure	2.7	29.6	42.5	<0.001
Chronic pulmonary disease	10.5	27.4	25.5	<0.001
Cancer	2.0	9.9	8.8	<0.001
Hospital region, %				<0.001
Northeast	19.1	17.7	18.8	
Midwest	22.2	22.8	24.0	
South	40.4	40.7	38.2	
West	18.3	18.8	19.0	
Location/teaching status of hospital, %				<0.001
Rural	26.6	25.4	24.4	
Urban non‐teaching	56.9	62.9	66.5	
Urban teaching	16.5	11.7	9.1	
Length of stay (days), median (IQR)	3 (2, 5)	4 (3, 7)	7 (4, 11)	<0.001
Total ED and in‐hospital charges (USD), median (IQR)	24 986 (14 613, 44 245)	35 453 (20 277, 65 826)	54 428 (29 438, 105 862)	<0.001
Disposition, %				<0.001
Home	80.4	17.0	1.3	
Transfer to short‐term hospital	2.1	1.7	0.3	
Other transfer	1.6	1.6	0.8	
Home health care	0.2	0.7	0.4	
Discharge against medical advice	1.3	0.4	<0.1	
Admitted as inpatient	14.0	78.4	97.1	
ED all‐cause mortality, %	0.4	0.2	0.1	<0.001
Overall all‐cause mortality, %	0.4	3.7	9.6	<0.001

AMI, acute myocardial infarction; CABG, coronary artery bypass graft; ED, emergency department; HFRS, Hospital Frailty Risk Score; IQR, interquartile range; PCI, percutaneous coronary intervention; USD, United States dollar.

**Table 2:** Cause of admission stratified by HFRS

Admission diagnosis		Hospital Frailty Risk Score			*P*‐value
		Low <5 (80.9%)	Intermediate 5–15 (17.5%)	High >15 (1.6%)	
Infectious diseases (*N* = 11 201 562)	Prevalence	4.5	16.6	43.0	<0.001
	ED mortality	0.1	0.1	0.1	<0.001
	Overall mortality	0.2	8.0	12.7	<0.001
Neoplasms (*N* = 2 110 162)	Prevalence	1.0	2.9	1.8	<0.001
	ED mortality	0.2	0.1	<0.1	<0.001
	Overall mortality	1.4	8.8	14.5	<0.001
Hematological diseases (*N* = 2 296 711)	Prevalence	1.4	1.7	0.8	<0.001
	ED mortality	<0.1	<0.1	<0.1	<0.001
	Overall mortality	0.1	1.6	5.2	<0.001
Endocrine diseases (*N* = 7 862 684)	Prevalence	4.0	9.9	5.5	<0.001
	ED mortality	<0.1	<0.1	<0.1	<0.001
	Overall mortality	<0.1	0.7	3.4	<0.001
Neurological diseases (*N* = 9 921 873)	Prevalence	6.6	5.6	5.2	<0.001
	ED mortality	<0.1	<0.1	<0.1	<0.001
	Overall mortality	<0.1	1.0	3.5	<0.001
Cardiovascular diseases (*N* = 20 498 940)	Prevalence	10.7	23.5	24.0	<0.001
	ED mortality	3.1	0.4	0.1	<0.001
	Overall mortality	3.5	4.3	8.4	<0.001
Respiratory diseases (*N* = 30 306 088)	Prevalence	20.6	15.4	10.2	<0.001
	ED mortality	<0.1	0.2	0.1	<0.001
	Overall mortality	0.1	4.1	9.9	<0.001
Gastrointestinal diseases (*N* = 27 035 737)	Prevalence	18.5	13.2	5.9	<0.001
	ED mortality	<0.1	<0.1	0.1	<0.001
	Overall mortality	0.1	2.3	7.4	<0.001
Musculoskeletal diseases (*N* = 28 537 858)	Prevalence	21.2	6.6	2.1	<0.001
	ED mortality	<0.1	<0.1	<0.1	<0.001
	Overall mortality	<0.1	0.3	2.6	<0.001
Psychiatric diseases (*N* = 15 725 433)	Prevalence	11.5	4.6	1.4	<0.001
	ED mortality	<0.1	<0.1	<0.1	<0.001
	Overall mortality	<0.1	0.2	1.5	<0.001

ED, emergency department; HFRS, Hospital Frailty Risk Score.

Attendances with a high HFRS had a higher prevalence of comorbidities, such as dyslipidaemia, thrombocytopaenia, anaemias, peripheral vascular disorders, diabetes, coagulopathy, and chronic renal failure, compared with patients with an intermediate and low HFRS, respectively (*P* < 0.001 for all). Furthermore, high‐HFRS attendances had an increased length of stay and total costs compared with patients with an intermediate and low HFRS (Table 1).

**Figure 1:**
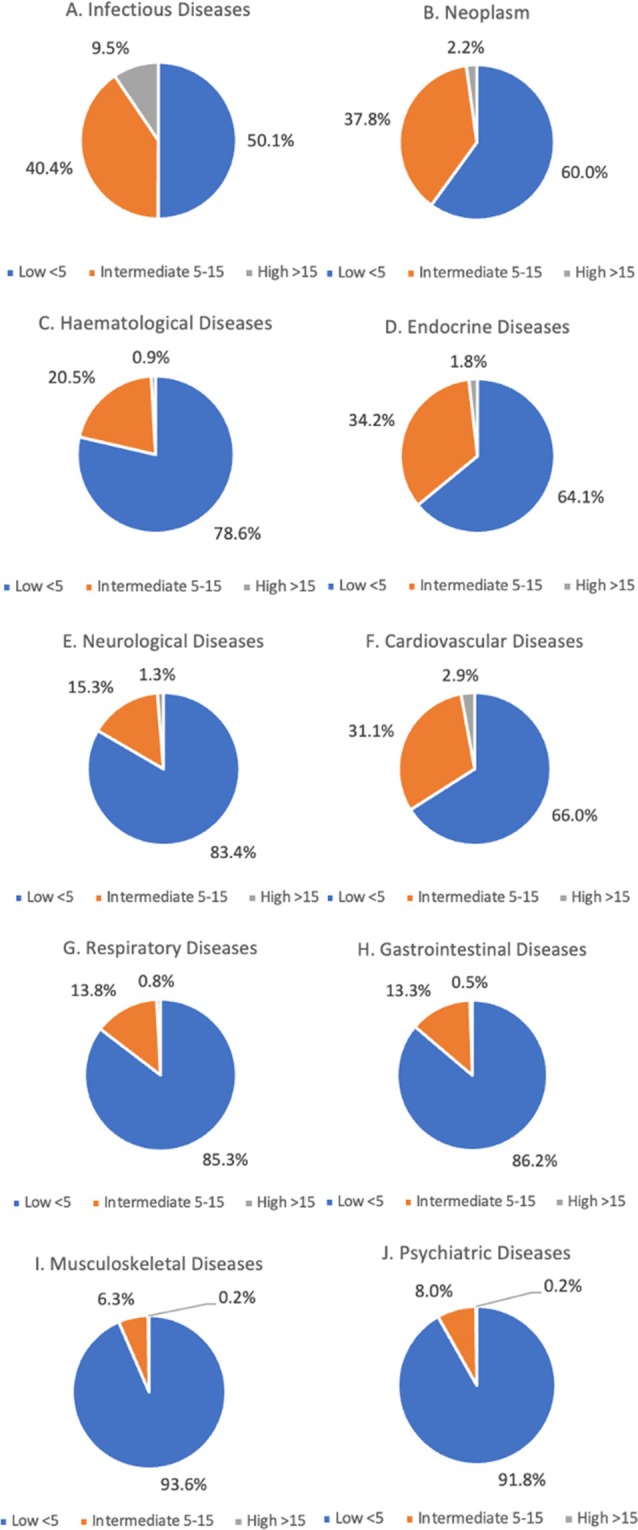
Distribution of each HFRS category within each of the selected ED cardiovascular admission causes. ED, emergency department; HFRS, Hospital Frailty Risk Score.

The distribution of low, intermediate, and high HFRS varied according to the attendance cause. The highest proportion of attendances with high HFRS was observed in the cohort admitted with infectious diseases (9.5%), followed by the CVD (2.9%) and neoplasm (2.2%) cohorts. Similarly, the infectious diseases cohort also had the highest proportion of attendances with an intermediate HFRS (40.4%), followed by the neoplasm (37.8%) and endocrine diseases (34.2%) cohorts. The cohort admitted with musculoskeletal diseases had the highest proportion of attendances with a low HFRS (93.6%), followed by the psychiatric diseases (91.8%) and gastrointestinal diseases (86.2%) cohorts (Fig. 1).

The distribution of each cause of attendance varied according to frailty status. In the high‐HFRS group, infectious diseases were the most common cause of ED attendance (43.0%), followed by CVD (24.0%) and respiratory diseases (10.2%). For the intermediate‐HFRS group, CVD were the most common cause of ED attendance (23.5%), followed by infectious diseases (16.6%) and respiratory diseases (15.4%). The most common cause of ED attendance in the low‐HFRS group was musculoskeletal diseases (21.2%), followed by respiratory diseases (20.6%) and gastrointestinal diseases (18.5%) (Table 2 and Fig. S2).

### Characteristics stratified by attendance causes

Attendances for infectious diseases with a high HFRS were more likely to be older and female compared with their intermediate‐ or low‐HFRS counterparts. These patients also had more comorbid conditions, such as dyslipidaemia, thrombocytopaenia, anemia, and valvular heart diseases, compared with their low‐HFRS counterparts (*P* < 0.001). Similar findings were observed amongst attendances for neoplasms, hematological diseases, endocrine diseases, CVD, gastrointestinal diseases, musculoskeletal diseases, and psychiatric diseases (Tables S2–S11).

### Discharge disposition and all‐cause mortality

Attendances with a high HFRS were more likely to be admitted as an inpatient and less likely to be transferred to a short‐term hospital (*P* < 0.001) (Table 1). Interestingly, attendances with a high HFRS generally had lower unadjusted rates of ED all‐cause mortality compared with attendances with a lower frailty score (*P* < 0.001). However, high HFRS was associated with increased rates of overall mortality (combined ED and in‐hospital mortality) (*P* < 0.001). This trend was observed across all attendance causes (Table 2).

**Figure 2:**
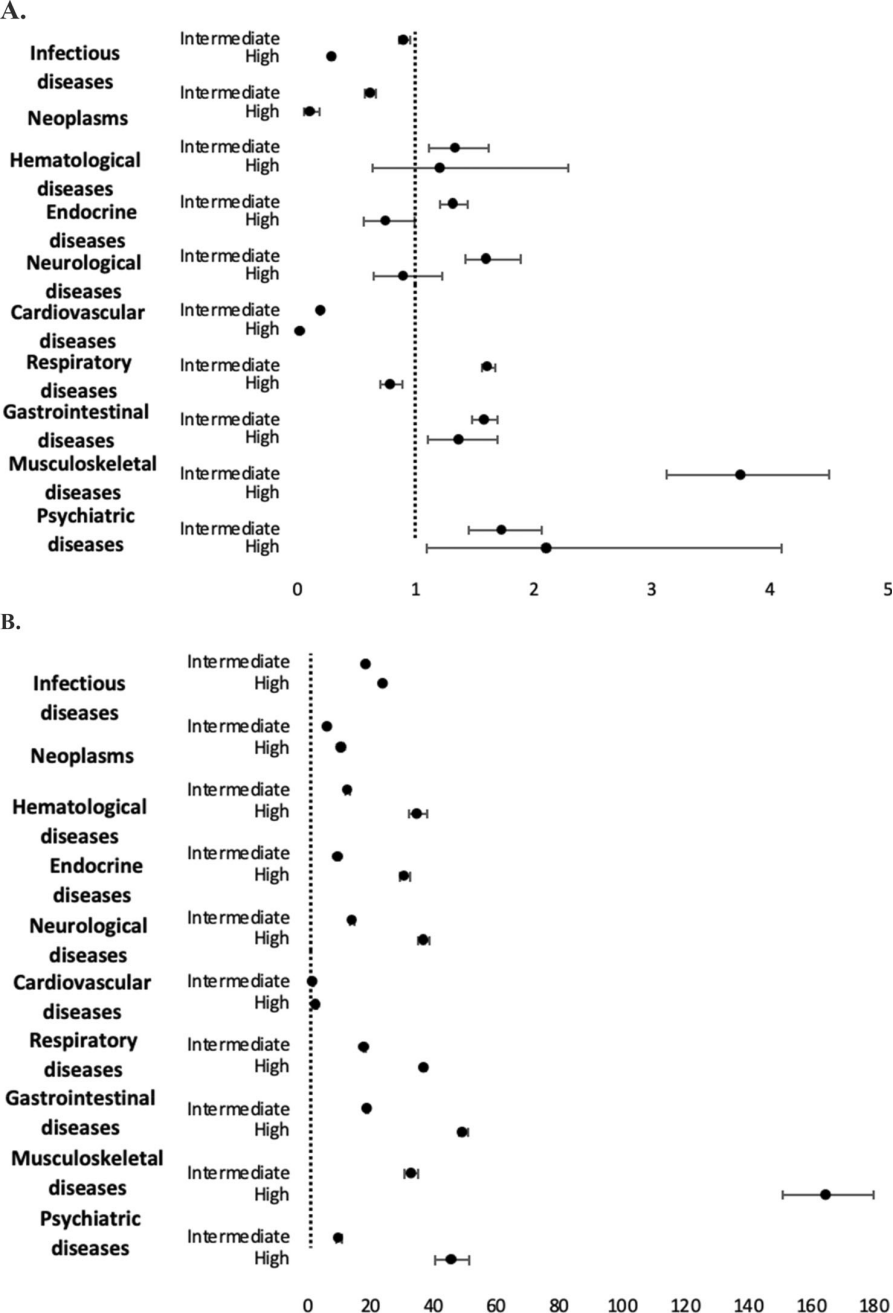
Adjusted mortality rates for different frailty risk categories and admission cause* for (a) ED mortality and (b) overall mortality. *Reference group is low HFRS score <5 for each admission diagnosis. Multivariable logistic regression model adjusted for: age, sex, weekend admission, primary expected payer, median household income, region and teaching status of the hospital, dyslipidemia, smoking, previous AMI, coagulopathies, chronic renal disease, and valvular heart diseases. AMI, acute myocardial infarction; aOR, adjusted odds ratio; CI, confidence interval; ED, emergency department; HFRS, hospital frailty risk score.

On adjustment for baseline covariates, the high‐HFRS groups admitted with gastrointestinal diseases and psychiatric diseases had an increased odds of both ED and overall mortality compared with their low‐frailty‐risk counterparts (*P* < 0.001). On the other hand, the high‐HFRS groups admitted with infectious diseases, neoplasms, CVD, and respiratory diseases had a decreased odds of ED mortality compared with patients with a low frailty risk score (*P* < 0.001). Nevertheless, intermediate and high HFRS was associated with an increased odds of overall all‐cause mortality across all attendance categories (*P* < 0.001). When looking at the effect size, attendances with high HFRS admitted with psychiatric diseases had the highest odds of ED mortality, compared with their low‐frailty‐risk counterparts (Table 3 and Figure 2).

**Table 3:** Adjusted odds of ED mortality by admission cause stratified by HFRS^†^

Admission diagnosis		Hospital Frailty Risk Score			
		Intermediate 5–15 (17.5%)		High >15 (1.6%)	
		aOR	*P‐*value	aOR	*P‐*value
Infectious diseases (*N* = 11 201 562)	Hospitalisation	51.33 [51.06–51.60]	<0.001	239.94 [235.03–244.96]	<0.001
	ED mortality	0.90 [0.86–0.95]	<0.001	0.29 [0.27–0.32]	<0.001
	Overall mortality	18.50 [18.16–18.84]	<0.001	23.88 [23.42–24.34]	<0.001
Neoplasms (*N* = 2 110 162)	Hospitalisation	10.77 [10.66–10.88]	<0.001	60.03 [54.93–65.61]	<0.001
	ED mortality	0.62 [0.57–0.66]	<0.001	0.11 [0.06–0.19]	<0.001
	Overall mortality	6.18 [6.07–6.29]	<0.001	10.65 [10.31–10.99]	<0.001
Hematological diseases (*N* = 2 296 711)	Hospitalisation	7.08 [7.02–7.15]	<0.001	38.24 [35.83–40.81]	<0.001
	ED mortality	1.34 [1.11–1.62]	0.002	1.21 [0.64–2.29]	0.553
	Overall mortality	12.75 [12.03–13.51]	<0.001	34.84 [32.00–37.93]	<0.001
Endocrine diseases (*N* = 7 862 684)	Hospitalisation	9.09 [9.05–9.13]	<0.001	52.10 [51.10–53.11]	<0.001
	ED mortality	1.32 [1.21–1.44]	<0.001	0.75 [0.56–0.99]	0.030
	Overall mortality	9.63 [9.22–10.06]	<0.001	30.92 [29.34–32.58]	<0.001
Neurological diseases (*N* = 9 921 873)	Hospitalisation	10.80 [10.74–10.85]	<0.001	51.50 [50.52–52.50]	<0.001
	ED mortality	1.60 [1.42–1.80]	<0.001	0.90 [0.65–1.23]	0.498
	Overall mortality	14.10 [13.53–14.67]	<0.001	36.88 [35.06–38.81]	<0.001
Cardiovascular diseases (*N* = 20 498 940)	Hospitalisation	7.50 [7.48–7.53]	<0.001	51.38 [50.56–52.22]	<0.001
	ED mortality	0.20 [0.19–0.20]	<0.001	0.03 [0.02–0.03]	<0.001
	Overall mortality	1.38 [1.37–1.38]	<0.001	2.58 [2.55–2.61]	<0.001
Respiratory diseases (*N* = 30 306 088)	Hospitalisation	23.85 [23.77–23.93]	<0.001	146.43 [142.57–150.38]	<0.001
	ED mortality	1.61 [1.56–1.67]	<0.001	0.79 [0.70–0.89]	<0.001
	Overall mortality	18.15 [17.89–18.41]	<0.001	36.90 [36.18–37.62]	<0.001
Gastrointestinal diseases (*N* = 27 035 737)	Hospitalisation	9.98 [9.95–10.01]	<0.001	62.88 [60.93–64.90]	<0.001
	ED mortality	1.58 [1.48–1.69]	<0.001	1.36 [1.10–1.69]	0.005
	Overall mortality	18.86 [18.48–19.24]	<0.001	49.36 [47.97–50.79]	<0.001
Musculoskeletal diseases (*N* = 28 537 858)	Hospitalisation	12.04 [11.99–12.10]	<0.001	124.39 [121.11–127.75]	<0.001
	ED mortality	3.75 [3.12–4.50]	<0.001	n/a^‡^	n/a^‡^
	Overall mortality	32.76 [30.61–35.06]	<0.001	164.76 [150.83–179.98]	<0.001
Psychiatric diseases (*N* = 15 725 433)	Hospitalisation	7.65 [7.62–7.69]	<0.001	46.28 [44.48–48.14]	<0.001
	ED mortality	1.73 [1.45–2.07]	<0.001	2.11 [1.09–4.10]	0.028
	Overall mortality	9.94 [9.20–10.74]	<0.001	45.62 [40.50–51.38]	<0.001

Multivariable logistic regression model adjusted for: age, sex, weekend admission, primary expected payer, median household income, region and teaching status of the hospital, dyslipidemia, smoking, previous AMI, coagulopathies, chronic renal disease and valvular heart diseases. AMI, acute myocardial infarction; aOR, adjusted odds ratio; CI, confidence interval; ED, emergency department; HFRS, Hospital Frailty Risk Score.

^†^ Reference group is low HFRS score <5 for each admission diagnosis.

^‡^ Results could not be reported owing to low number of cases.

With the HFRS modeled as a continuous variable, increased HFRS was associated with significantly higher odds of hospitalization and ED and overall mortality across most attendance causes per 1‐unit increase of the HFRS (all *P* < 0.001), except amongst infectious diseases mortality, neoplasms and CVD which showed a lower odds of ED. There was no difference in ED mortality in attendances with endocrine diseases (Table S12).

### Most common diagnoses based on each ED admission cause group stratified by HFRS

Among the most common causes of admission in the high‐HFRS group, sepsis was the most common cause of attendance in the infectious disease group (31.2%), secondary malignant neoplasm of the brain was the most common cause for the neoplasm group (0.1%), acute post‐hemorrhagic anemia was the most common cause in the hematological diseases group (0.2%), dehydration was the most common cause in the endocrine diseases group (1.0%), transient ischemic attack was the most common cause in the neurological diseases group (0.7%), cerebral infarction was the most common cause in the CVD group (4.2%), aspiration from food was the most common cause in the respiratory diseases group (2.5%), gastrointestinal hemorrhage was the most common cause in the gastrointestinal diseases group (0.6%), rhabdomyolysis was the most common cause in the musculoskeletal diseases group (0.3), and alcohol withdrawal was the most common cause in the psychiatric diseases group (0.2%) (Table S13).

### Analysis for patients aged over 65

When restricting the cohort to attendances in patients aged over 65 only, there were a total of 42 112 989 patients (27.1% of the original cohort). Of them, 24 856 421 (59.0%) had a low HFRS, with 15 374 744 (36.5%) having an intermediate HFRS and the remaining 1 881 824 (4.5%) having a high HFRS. Attendances with a high HFRS were more likely to be older than their intermediate‐ and low‐HFRS counterparts (median age 81 vs. 68 for intermediate and 76 for low HFRS). High‐HFRS attendances were more likely to be more comorbid with conditions such as thrombocytopaenia, anemias, peripheral vascular disorders, coagulopathy, diabetes, and chronic renal failure (*P* < 0.001 for all). High‐HFRS attendances had a longer length of stay, higher total charges, and were more likely to be admitted as an inpatient compared with their intermediate‐ and low‐HFRS counterparts (*P* < 0.001). Although high‐HFRS attendances had a lower likelihood of ED mortality, they had a higher likelihood of combined ED and in‐hospital mortality compared with their intermediate‐ and low‐HFRS counterparts (*P* < 0.001) (Table S14).

On adjusted analysis for attendances in attendances in those over the age of 65, across all admission groups, intermediate and high HFRS were associated with increased odds of hospitalisation and overall mortality, and with lower odds of ED mortality, compared with their low‐HFRS counterparts (all *P* < 0.001). When looking at effect size, high‐HFRS attendances admitted with musculoskeletal and conditions had the highest odds of overall mortality compared with their low‐HFRS counterparts (Table S15).

## Discussion

To the best of our knowledge, this is the largest study to examine the prevalence, causes, clinical characteristics, and mortality of patients attending ED with a broad range of acute presentations stratified by frailty status. We report several important findings. First, intermediate and high frailty risk is present in a significant proportion of ED attendances, with the distribution of attendance cause differing according to frailty status. Second, higher frailty risk is associated with increasing age, female sex, comorbidities, length of stay, and total cost. Third, high‐HFRS patients are more likely to be admitted as an inpatient and less likely to be discharged home across most attendance causes. Finally, increasing HFRS is associated with a higher risk of mortality across most causes of ED attendance.

### Prevalence of frailty in emergency department cohorts

The reported prevalence of frailty in patients admitted to EDs varies in the literature, as there is no agreed definition or tool to assess frailty.^13^ Previous studies have used various tools to assess the prevalence of frailty in the ED; however, many of these studies are restricted to specific populations and do not use the HFRS. The present study found that 80.9% of ED patients had a low HFRS, 17.5% had an intermediate HFRS, and 1.6% had a high HFRS. A similar distribution of frailty risk was reflected in a study on a Swiss ED cohort: 63.5% were low risk, 33.5% were intermediate risk, and 2.9% were high risk for frailty.^14^ However, a study using the HFRS in a French ED cohort found a prevalence of an intermediate and high risk of frailty 37.1% and 17.5%, respectively.^15^ This contrasting finding could be due to a restricted inclusion criteria of patients over 75, highlighting the importance of frailty in younger patient groups.

### Demographics of frail patients presenting to the emergency department

Increasing HFRS was associated with increasing age, female sex, multimorbidity, and mortality.^15^ In 12 237 patients over 75 from a single centre in the UK study, 47.9% had an intermediate risk of frailty and 17.5% had a high risk of frailty.^16^ Increasing frailty was associated with increased age, female sex, more comorbidities, and mortality.^16^ However, again, the selection criteria neglected the increasingly young frail population described. The present study observed a lower proportion of intermediate‐risk (17.5%) and high‐risk (1.6%) patients yet a similar trend in clinical characteristics. This could be explained by the lower age and the broader inclusion criteria of adults in the present study, yielding a more representative burden of frailty in an unfiltered adult ED setting.^17^ In our analysis of over‐65 year‐olds only, 59.0% had a low HFRS, 36.5% had an intermediate HFRS, and 4.5% had a high HFRS, demonstrating a higher burden of frailty with increasing age.

### Distribution of frailty status across admission causes

This study reported variations in frailty status across different attendance causes. Patients admitted with infectious diseases, neoplasms, endocrine diseases, and CVD had the highest proportions of patients with an intermediate‐ or high‐risk HFRS. Patients with these diagnoses are usually older and more comorbid, with both of these conditions linked with frailty.^18^ Frail patients could be more susceptible to infectious diseases owing to dysregulated inflammation, a weakened immune system, and decreased clinical response to vaccines.^19^ Additionally, frail patients or caregivers may have a lower threshold for seeking healthcare, particularly if they experience acute illness.^20^ It is unsurprising that cancer was commonly observed in high‐ and intermediate‐risk frailty patients. Previous work suggests that frailty is common in cancer patients such as those with lung (45%) and breast (43%) cancer.^21^, ^22^ Cancer patients are multimorbid, with around 25% of colorectal and lung cancer patients having at least two comorbidities.^23^ Multimorbidity can lead to functional decline, malnutrition, sarcopenia, and weight loss, exacerbating frailty.^24^, ^25^, ^26^ Studies have also demonstrated that frailty is present in a significant proportion of CVD patients, with up to 40% at either intermediate or high risk of frailty.^8^, ^27^ Frailty and CVD have a bidirectional relationship owing to the underlying mechanism of chronic inflammation.^28^ Clinical studies about frailty and endocrine diseases focus on diabetic patients, neglecting other endocrine diseases. For instance, thyroid diseases are considered conditions of older age and frailty.^29^ Interestingly, in the present study, musculoskeletal disorders were the most common cause of admission in the low‐HFRS group. This could be due to younger patients with low frailty risk being more likely to present to ED with minor sports or trauma‐related musculoskeletal injuries.

### ED mortality rates in frail patients

The study showed that absolute risk of ED mortality was low, and, therefore, the observed effect size for ED mortality is lower than that of overall mortality. This varied across diagnostic categories. However, increasing frailty was also associated with a decreased ED mortality across most attendance causes. This could be due to early identification, triaging and referral for management of patients with higher frailty risk, leading to a lower likelihood of adverse outcomes in the ED setting, but with an overall worse prognosis during their in‐hospital stays. This was demonstrated in the present study, where overall mortality increased across all attendance causes, similar to in a previous NEDS study.^8^ Although it is hard to delineate the exact mechanisms underlying the present findings, it should be noted that in this study, frail patients were less likely to be discharged home and more likely to be hospitalised and have a higher in‐hospital mortality. Therefore, adverse events could have occurred when patients were hospitalised and not when patients were in the ED.

### Clinical implications

There are several important clinical implications of this study. This study prompts the early recognition and management of frailty in the ED setting, given that frailty is present in a substantial proportion of the ED population. Whether frailty assessments in the ED could lead to improved outcomes is unclear,^30^, ^31^ but early recognition of frail patients may allow clinicians to detect patients at risk.^32^, ^33^, ^34^ Management of frailty can occur in hospitalised patients and in the community, which can impact long‐term outcomes.^35^ The HFRS could flag patients at higher risk of frailty directly from comorbidities from their electronic health records, where their risk factors can be optimised. Overall, knowledge of the trends in frailty and reasons for ED attendance is important, so healthcare infrastructure can be built around the needs of the growing frail population.

## Limitations

There are limitations of this study inherent to the NEDS dataset. First, electronic health record databases are susceptible to selection bias owing to miscoding and missing data. Second, clinical information such as race and the pharmacological management of patients are not available in the NEDS. Third, this analysis was based on data from the United States, so may not be generalisable to other countries and healthcare infrastructures. Fourth, it was not possible to avoid collinearity between age and HFRS in our multivariable models. Finally, the NEDS captures only hospital outcomes, and therefore longitudinal outcomes could not be assessed.

## Conclusions

In conclusion, the cause of attendance to EDs varies by frailty status. The infectious diseases cohort had the highest proportion of patients with high and intermediate HFRS, whereas patients with a low HFRS were the most common patients attending with musculoskeletal disorders. The association of frailty and ED mortality depends on the underlying cause of attendance, and increasing frailty is associated with an overall worse short‐term prognosis across most diagnostic categories. In the future, more granular studies are required to investigate the longitudinal association between frailty and mortality in the ED.

## Funding information

The authors received no funding for this study. Christian D. Mallen is funded by the National Institute for Health Research (NIHR) Applied Research Collaboration West Midlands and the NIHR School for Primary Care Research.

## Disclosure statement

The authors declare no conflict of interest.

## Author contributions

**Conceptualisation:** Balamrit Singh Sokhal, Andrija Matetić, Mamas A. Mamas; **Methodology:** Balamrit Singh Sokhal, Andrija Matetić; **Formal analysis:** Balamrit Singh Sokhal, Andrija Matetić; **Writing – original draft:** Balamrit Singh Sokhal, Andrija Matetić, Christian D. Mallen, Mamas A. Mamas; **Writing – review and editing:** Balamrit Singh Sokhal, Andrija Matetić, Joanne Protheroe, Toby Helliwell, Phyo K. Myint, Timir K. Paul, Christian D. Mallen, Mamas A. Mamas; **Visualisation:** Balamrit Singh Sokhal, Andrija Matetić; **Supervision:** Andrija Matetić, Christian D. Mallen, Mamas A. Mamas; **Project administration:** Mamas A. Mamas.

## Supporting information


**Table S1.** ICD‐10 codes used in the study.
**Table S2.** Characteristics of patients presenting with infectious diseases.
**Table S3.** Characteristics of patients presenting with neoplasms.
**Table S4.** Characteristics of patients presenting with hematological diseases.
**Table S5.** Characteristics of patients presenting with endocrine diseases.
**Table S6.** Characteristics of patients presenting with neurological diseases.
**Table S7.** Characteristics of patients presenting with cardiovascular diseases.
**Table S8.** Characteristics of patients presenting with respiratory diseases.
**Table S9.** Characteristics of patients presenting with gastrointestinal diseases.
**Table S10.** Characteristics of patients presenting with musculoskeletal diseases.
**Table S11.** Characteristics of patients presenting with psychiatric diseases.
**Table S12.** Adjusted odds of ED mortality by admission cause stratified by HFRS modelled as a continuous variable*.
**Table S13.** Most common diagnoses from each cause of ED encounter stratified by HFRS*.
**Table S14.** Characteristics of patients over 65 presenting to the ED.
**Table S15.** Adjusted odds of ED mortality by admission cause stratified by HFRS in patients aged over 65 only*.
**Figure S1.** Flow diagram of the cohort selection process.
**Figure S2.** Distribution of cause of admission within each HFRS category.
**Appendix S1.** Components of Hospital Frailty Risk Score with associated weighting.
**Appendix S2.** STROBE (Strengthening The Reporting of OBservational Studies in Epidemiology) checklist.

## Data Availability

These data were derived from the following resources available in the public domain: HCUP, https://hcup-us.ahrq.gov.
