# Supplementary material for: Emergency department attendance stratified by cause and frailty status: A national retrospective cohort study
Source: Geriatr Gerontol Int. 2025 Aug 28;25(10):1350–8. doi: 10.1111/ggi.70153 (PMC12501700; doi:10.1111/ggi.70153)
Supplement: Supplementary file 1 — Table S1. ICD‐10 codes used in the study. Table S2. Characteristics of patients presenting with infectious diseases. Table S3. Characteristics of patients presenting with neoplasms. Table S4. Characteristics of patients presenting with hematological diseases. Table S5. Characteristics of patients presenting with endocrine diseases. Table S6. Characteristics of patients presenting with neurological diseases. Table S7. Characteristics of patients presenting with cardiovascular diseases. Table S8. Characteristics of patients presenting with respiratory diseases. Table S9. Characteristics of patients presenting with gastrointestinal diseases. Table S10. Characteristics of patients presenting with musculoskeletal diseases. Table S11. Characteristics of patients presenting with psychiatric diseases. Table S12. Adjusted odds of ED mortality by admission cause stratified by HFRS modelled as a continuous variable*. Table S13. Most common diagnoses from each cause of ED encounter stratified by HFRS*. Table S14. Characteristics of patients over 65 presenting to the ED. Table S15. Adjusted odds of ED mortality by admission cause stratified by HFRS in patients aged over 65 only*. Figure S1. Flow diagram of the cohort selection process. Figure S2. Distribution of cause of admission within each HFRS category. Appendix S1. Components of Hospital Frailty Risk Score with associated weighting. Appendix S2. STROBE (Strengthening The Reporting of OBservational Studies in Epidemiology) checklist. [file GGI-25-1350-s001.docx]

**Supplementary Tables and Figures**

**Supplementary Table 1.** ICD-10 codes used in the study.

| **ICD-10 Chapters** | **Source** | **Codes** |
| --- | --- | --- |
| **Infectious diseases** | ICD-10 | A00-B99 |
| **Neoplasm** | ICD-10 | C00-D48 |
| **Haematological diseases** | ICD-10 | D50-D89 |
| **Endocrine diseases** | ICD-10 | E00-E90 |
| **Neurological diseases** | ICD-10 | G00-G99 |
| **Cardiovascular diseases** | ICD-10 | I00-I99 |
| **Respiratory diseases** | ICD-10 | J00-J99 |
| **Gastrointestinal diseases** | ICD-10 | K00-K93 |
| **Musculoskeletal diseases** | ICD-10 | M00-M99 |
| **Psychiatric diseases** | ICD-10 | F00-F99 |
| **Variables** | **Source** | **Codes** |
| **Acute ischemic stroke** | ICD-10 | I63* |
| **Acute haemorrhagic stroke** | ICD-10 | 430, 431, 432.0, 432.1, 432.9 |
| **Heart Failure** | ICD-10 | I50* Cardiomyopathy, I42* |
| **AF** | ICD-10 | I48.91, I48.20-21, I48.11, I48.19, I4.80 |
| **Cardiac arrest** | ICD-10 | I46.2 (due to cardiac condition); I46.8 and I46.9 (due to non-cardiac condition) |
| **Acute MI** | ICD-10 | 410.0x, 410.1x, 410.2x, 410.3x, 410.4x, 410.5x, 410.6x, 410.7x, 410.8x, 410.9x |
| **Pulmonary embolism** | ICD-10 | I26 |
| **Dyslipidaemia** | ICD-10 | E78* |
| **Smoker** | ICD-10 | Z72.0 |
| **Previous MI** | ICD-10 | I25.2, I25.6 |
| **Previous PCI** |  | Z98.61, Z95.5 |
| **Previous CABG** |  | Z95.1, Z95.5 |
| **Thrombocytopenia** | ICD-10 | D69.4*, D69.5*, D69.6* |
| **Homelessness** | ICD-10 | Z59.0 |
| **Chronic renal failure** | ICD-10 | N18* |
| **Hypertension** | ICD-10 | I10* |
| **Anemias** | ICD-10 | D62*, D63*, D64* |
| **Diabetes Mellitus** | ICD-10 | E08*, E09*, E10*, E11*, E13* |
| **Coagulopathies** | ICD-10 | D65, D66, D67, D68*, D69* |
| **Liver disease** | ICD-10 | K70*, K72.1*, K72.9*, K73*, K74*, K75*, K76*, K77* |
| **Metastatic disease** | ICD-10 | C77*, C78*, C79*, R18.0*, C7B* |
| **PVD** | ICD-10 | I73* |
| **Valvular heart disease** | ICD-10 | I34*, I35*, I36*, I37* |

**Abbreviations:** AF: atrial fibrillation; CABG – coronary artery bypass grafting; CVA – cerebrovascular accident; HF – Heart Failure; ICD-10 – International Classification of Diseases Tenth Edition; MI – myocardial infarction; PCI: percutaneous coronary intervention; PVD – peripheral vascular disease; TIA: transient ischemic attack.

**Supplementary Table 2.** Characteristics of patients presenting with infectious diseases.

| **Characteristics** | **Hospital Frailty Risk Score** | | | ***P*-value** |
| --- | --- | --- | --- | --- |
|  | **Low <5**  **(50.1%)** | **Intermediate 5-15**  **(40.4%)** | **High >15**  **(9.5%)** |  |
| **Number of weighted discharges** | 5,610,599 | 4,522,868 | 1,068,095 |  |
| **Age (years), median (IQR)** | 52 (36,67) | 67 (55, 78) | 76 (66, 85) | <0.001 |
| **Female sex, %** | 59.0 | 51.2 | 53.6 | <0.001 |
| **Weekend admission, %** | 28.1 | 26.8 | 26.4 | <0.001 |
| **Primary expected payer, %** |  |  |  | <0.001 |
| **Medicare** | 18.9 | 62.3 | 80.1 |  |
| **Medicaid** | 30.1 | 14.2 | 8.7 |  |
| **Private Insurance** | 29.9 | 17.3 | 8.4 |  |
| **Self-pay** | 17.4 | 3.9 | 1.3 |  |
| **No charge** | 0.6 | 0.3 | 0.1 |  |
| **Other** | 3.1 | 1.9 | 1.4 |  |
| **Median Household Income (percentile), %** |  |  |  | <0.001 |
| **0-25^th^** | 38.7 | 31.1 | 30.5 |  |
| **26^th^–50^th^** | 27.2 | 26.5 | 25.2 |  |
| **51^st^-75^th^** | 19.4 | 22.5 | 22.4 |  |
| **76^th^–100^th^** | 14.7 | 19.9 | 21.8 |  |
| **Comorbidities, %** |  |  |  |  |
| **Dyslipidaemia** | 7.0 | 36.1 | 39.9 | <0.001 |
| **Thrombocytopenia** | 1.0 | 10.1 | 12.1 | <0.001 |
| **Smoking** | 13.3 | 13.7 | 6.5 | <0.001 |
| **Previous AMI** | 0.9 | 5.9 | 6.0 | <0.001 |
| **Previous PCI** | 0.7 | 5.3 | 4.4 | <0.001 |
| **Previous CABG** | 0.6 | 5.1 | 4.8 | <0.001 |
| **Anemias** | 3.4 | 32.9 | 43.1 | <0.001 |
| **Valvular disease** | 0.4 | 5.8 | 7.4 | <0.001 |
| **Peripheral vascular disorders** | 0.1 | 3.5 | 4.7 | <0.001 |
| **Coagulopathy** | 1.2 | 12.9 | 15.6 | <0.001 |
| **Diabetes Mellitus** | 10.2 | 37.2 | 41.0 | <0.001 |
| **Liver disease** | 1.3 | 8.1 | 6.7 | <0.001 |
| **Chronic renal failure** | 1.4 | 25.0 | 42.6 | <0.001 |
| **Chronic pulmonary disease** | 4.2 | 26.1 | 25.5 | <0.001 |
| **Cancer** | 1.6 | 11.9 | 8.8 | <0.001 |
| **Hospital Region, %** |  |  |  | <0.001 |
| **Northeast** | 20.2 | 18.2 | 19.3 |  |
| **Midwest** | 20.9 | 20.5 | 22.2 |  |
| **South** | 40.9 | 38.1 | 36.9 |  |
| **West** | 18.0 | 23.2 | 21.6 |  |
| **Location/teaching status of hospital, %** |  |  |  | <0.001 |
| **Rural** | 26.1 | 27.0 | 26.6 |  |
| **Urban non-teaching** | 57.6 | 62.2 | 64.1 |  |
| **Urban teaching** | 16.3 | 10.8 | 9.3 |  |
| **Length of stay (days), median (IQR)** | 3 (2, 5) | 5 (3, 8) | 7 (4, 12) | <0.001 |
| **Total ED and in-hospital charges (USD), median (IQR)** | 25,624 (15,840, 42,715) | 40,717 (22,822, 77,473) | 56,692 (30,585, 108,355) | <0.001 |
| **All-cause mortality, %** |  |  |  | <0.001 |
| **ED all-cause mortality** | 0.1 | 0.1 | 0.1 |  |
| **In-hospital all-cause mortality** | 0.2 | 7.8 | 12.6 |  |
| **Discharge disposition, %** |  |  |  | <0.001 |
| **Home** | 81.6 | 3.1 | 0.3 |  |
| **Transfer to short-term hospital** | 1.4 | 1.4 | 0.2 |  |
| **Other transfer** | 0.3 | 0.4 | 0.2 |  |
| **Home health care** | 0.1 | 0.2 | 0.2 |  |
| **Discharge against medical advice** | 0.5 | 0.1 | <0.1 |  |
| **Admitted as inpatient** | 16.0 | 94.7 | 99.1 |  |

**Abbreviations:** AMI – Acute Myocardial Infarction; CABG – Coronary Artery Bypass Graft; ED – Emergency Department; HFRS – Hospital Frailty Risk Score; IQR – Interquartile Range; PCI – Percutaneous Coronary Intervention; USD – United States Dollar.

**Supplementary Table 3.** Characteristics of patients presenting with neoplasms.

| **Characteristics** | **Hospital Frailty Risk Score** | | | ***P*-value** |
| --- | --- | --- | --- | --- |
|  | **Low <5**  **(60.0%)** | **Intermediate 5-15**  **(37.8%)** | **High >15**  **(2.2%)** |  |
| **Number of weighted discharges** | 1,265,979 | 798,543 | 45,641 |  |
| **Age (years), median (IQR)** | 63 (52, 73) | 69 (59, 78) | 73 (64, 82) | <0.001 |
| **Female sex, %** | 58.6 | 49.2 | 49.7 | <0.001 |
| **Weekend admission, %** | 23.0 | 23.1 | 23.1 | <0.001 |
| **Primary expected payer, %** |  |  |  | <0.001 |
| **Medicare** | 37.8 | 62.4 | 72.9 |  |
| **Medicaid** | 20.0 | 11.8 | 8.9 |  |
| **Private Insurance** | 29.9 | 20.7 | 14.7 |  |
| **Self-pay** | 9.1 | 2.9 | 1.9 |  |
| **No charge** | 0.6 | 0.2 | 0.1 |  |
| **Other** | 2.7 | 2.1 | 1.5 |  |
| **Median Household Income (percentile), %** |  |  |  | <0.001 |
| **0-25^th^** | 32.6 | 29.3 | 28.7 |  |
| **26^th^–50^th^** | 26.3 | 24.7 | 23.5 |  |
| **51^st^-75^th^** | 22.1 | 23.4 | 23.8 |  |
| **76^th^–100^th^** | 19.0 | 22.6 | 24.0 |  |
| **Comorbidities, %** |  |  |  |  |
| **Dyslipidaemia** | 16.9 | 35.5 | 38.9 | <0.001 |
| **Thrombocytopenia** | 3.8 | 10.1 | 12.8 | <0.001 |
| **Smoking** | 12.6 | 12.3 | 8.3 | <0.001 |
| **Previous AMI** | 2.3 | 5.3 | 5.1 | <0.001 |
| **Previous PCI** | 2.3 | 5.2 | 4.5 | <0.001 |
| **Previous CABG** | 1.7 | 4.2 | 3.8 | <0.001 |
| **Anemias** | 20.1 | 44.7 | 50.9 | <0.001 |
| **Valvular disease** | 1.2 | 4.2 | 5.5 | <0.001 |
| **Peripheral vascular disorders** | 0.3 | 1.3 | 1.9 | <0.001 |
| **Coagulopathy** | 4.6 | 13.2 | 17.1 | <0.001 |
| **Diabetes Mellitus** | 15.6 | 27.7 | 29.7 | <0.001 |
| **Liver disease** | 2.9 | 6.8 | 5.4 | <0.001 |
| **Chronic renal failure** | 3.3 | 20.7 | 37.5 | <0.001 |
| **Chronic pulmonary disease** | 9.4 | 22.0 | 20.9 | <0.001 |
| **Cancer** | 66.8 | 91.7 | 91.1 | <0.001 |
| **Hospital Region, %** |  |  |  | <0.001 |
| **Northeast** | 20.7 | 21.1 | 20.7 |  |
| **Midwest** | 17.5 | 20.4 | 22.6 |  |
| **South** | 43.4 | 41.9 | 41.0 |  |
| **West** | 18.4 | 37.8 | 15.6 |  |
| **Location/teaching status of hospital, %** |  |  |  | <0.001 |
| **Rural** | 23.5 | 20.8 | 19.7 |  |
| **Urban non-teaching** | 66.5 | 73.3 | 75.3 |  |
| **Urban teaching** | 9.9 | 5.9 | 5.1 |  |
| **Length of stay (days), median (IQR)** | 4 (2, 7) | 6 (3, 11) | 10 (6, 17) | <0.001 |
| **Total ED and in-hospital charges (USD), median (IQR)** | 41,698 (23,068, 75,789) | 56,481 (30,079, 108,305) | 84,008 (43,232, 165,348) | <0.001 |
| **All-cause mortality, %** |  |  |  | <0.001 |
| **ED all-cause mortality** | 0.2 | 0.1 | <0.1 |  |
| **In-hospital all-cause mortality** | 1.2 | 8.6 | 14.5 |  |
| **Discharge disposition, %** |  |  |  | <0.001 |
| **Home** | 47.0 | 4.8 | 0.4 |  |
| **Transfer to short-term hospital** | 2.9 | 0.6 | 0.1 |  |
| **Other transfer** | 1.2 | 0.7 | 0.3 |  |
| **Home health care** | 0.5 | 0.6 | 0.2 |  |
| **Discharge against medical advice** | 0.6 | <0.1 | <0.1 |  |
| **Admitted as inpatient** | 47.4 | 93.2 | 98.9 |  |

**Abbreviations:** AMI – Acute Myocardial Infarction; CABG – Coronary Artery Bypass Graft; ED – Emergency Department; HFRS – Hospital Frailty Risk Score; IQR – Interquartile Range; PCI – Percutaneous Coronary Intervention; USD – United States Dollar.

**Supplementary Table 4.** Characteristics of patients presenting with haematological diseases.

| **Characteristics** | **Hospital Frailty Risk Score** | | | ***P*-value** |
| --- | --- | --- | --- | --- |
|  | **Low <5**  **(78.6%)** | **Intermediate 5-15**  **(20.5%)** | **High >15**  **(0.9%)** |  |
| **Number of weighted discharges** | 1,804,756 | 471,198 | 20,757 |  |
| **Age (years), median (IQR)** | 47 (30, 67) | 69 (54, 80) | 77 (67, 85) | <0.001 |
| **Female sex, %** | 60.9 | 57.4 | 60.2 | <0.001 |
| **Weekend admission, %** | 23.2 | 21.9 | 22.5 | <0.001 |
| **Primary expected payer, %** |  |  |  | <0.001 |
| **Medicare** | 37.7 | 66.6 | 80.3 |  |
| **Medicaid** | 28.3 | 13.5 | 7.9 |  |
| **Private Insurance** | 23.4 | 15.2 | 9.1 |  |
| **Self-pay** | 7.8 | 2.7 | 1.2 |  |
| **No charge** | 0.4 | 0.2 | <0.1 |  |
| **Other** | 2.3 | 1.8 | 1.4 |  |
| **Median Household Income (percentile), %** |  |  |  | <0.001 |
| **0-25^th^** | 40.7 | 33.7 | 31.7 |  |
| **26^th^–50^th^** | 25.5 | 25.4 | 23.9 |  |
| **51^st^-75^th^** | 18.8 | 21.9 | 23.4 |  |
| **76^th^–100^th^** | 15.0 | 19.0 | 21.0 |  |
| **Comorbidities, %** |  |  |  |  |
| **Dyslipidaemia** | 10.9 | 36.1 | 42.1 | <0.001 |
| **Thrombocytopenia** | 7.5 | 12.8 | 13.6 | <0.001 |
| **Smoking** | 9.7 | 9.7 | 5.9 | <0.001 |
| **Previous AMI** | 2.1 | 7.3 | 7.8 | <0.001 |
| **Previous PCI** | 1.9 | 7.2 | 5.8 | <0.001 |
| **Previous CABG** | 1.7 | 6.6 | 6.2 | <0.001 |
| **Anemias** | 49.8 | 67.1 | 69.1 | <0.001 |
| **Valvular disease** | 1.3 | 6.8 | 10.4 | <0.001 |
| **Peripheral vascular disorders** | 0.3 | 2.3 | 3.4 | <0.001 |
| **Coagulopathy** | 11.0 | 21.8 | 29.3 | <0.001 |
| **Diabetes Mellitus** | 13.1 | 31.6 | 36.1 | <0.001 |
| **Liver disease** | 2.3 | 6.5 | 6.2 | <0.001 |
| **Chronic renal failure** | 6.4 | 34.7 | 50.2 | <0.001 |
| **Chronic pulmonary disease** | 6.3 | 20.4 | 23.1 | <0.001 |
| **Cancer** | 7.8 | 19.7 | 17.1 | <0.001 |
| **Hospital Region, %** |  |  |  | <0.001 |
| **Northeast** | 20.7 | 19.1 | 20.1 |  |
| **Midwest** | 17.9 | 22.7 | 25.3 |  |
| **South** | 46.2 | 42.4 | 39.3 |  |
| **West** | 15.2 | 15.8 | 15.3 |  |
| **Location/teaching status of hospital, %** |  |  |  | <0.001 |
| **Rural** | 23.6 | 24.0 | 24.3 |  |
| **Urban non-teaching** | 64.4 | 64.3 | 64.7 |  |
| **Urban teaching** | 12.0 | 11.7 | 11.0 |  |
| **Length of stay (days), median (IQR)** | 3 (2, 5) | 4 (2, 6) | 6 (4, 10) | <0.001 |
| **Total ED and in-hospital charges (USD), median (IQR)** | 23,092 (14,286, 38,640) | 31,760 (18,964, 55,446) | 46,071 (26,507, 84,191) | <0.001 |
| **All-cause mortality, %** |  |  |  | <0.001 |
| **ED all-cause mortality** | <0.1 | <0.1 | <0.1 |  |
| **In-hospital all-cause mortality** | 0.1 | 1.6 | 5.2 |  |
| **Discharge disposition, %** |  |  |  | <0.001 |
| **Home** | 62.8 | 16.0 | 1.8 |  |
| **Transfer to short-term hospital** | 3.1 | 2.0 | 0.4 |  |
| **Other transfer** | 1.8 | 2.7 | 1.8 |  |
| **Home health care** | 0.3 | 0.9 | 0.7 |  |
| **Discharge against medical advice** | 2.1 | 0.4 | <0.1 |  |
| **Admitted as inpatient** | 29.9 | 78.0 | 95.2 |  |

**Abbreviations:** AMI – Acute Myocardial Infarction; CABG – Coronary Artery Bypass Graft; ED – Emergency Department; HFRS – Hospital Frailty Risk Score; IQR – Interquartile Range; PCI – Percutaneous Coronary Intervention; USD – United States Dollar.

**Supplementary Table 5.** Characteristics of patients presenting with endocrine diseases.

| **Characteristics** | **Hospital Frailty Risk Score** | | | ***P*-value** |
| --- | --- | --- | --- | --- |
|  | **Low <5**  **(64.1%)** | **Intermediate 5-15**  **(34.2%)** | **High >15**  **(1.8%)** |  |
| **Number of weighted discharges** | 5,037,392 | 2,687,554 | 137,738 |  |
| **Age (years), median (IQR)** | 50 (33, 64) | 62 (50, 74) | 75 (64, 84) | <0.001 |
| **Female sex, %** | 53.0 | 52.8 | 59.5 | <0.001 |
| **Weekend admission, %** | 25.3 | 24.0 | 23.8 | <0.001 |
| **Primary expected payer, %** |  |  |  | <0.001 |
| **Medicare** | 34.8 | 58.1 | 78.7 |  |
| **Medicaid** | 23.9 | 17.1 | 8.9 |  |
| **Private Insurance** | 25.6 | 16.5 | 9.3 |  |
| **Self-pay** | 12.2 | 5.9 | 1.7 |  |
| **No charge** | 0.5 | 0.3 | 0.1 |  |
| **Other** | 2.9 | 2.1 | 1.4 |  |
| **Median Household Income (percentile), %** |  |  |  | <0.001 |
| **0-25^th^** | 38.4 | 36.4 | 32.6 |  |
| **26^th^–50^th^** | 27.8 | 26.7 | 25.2 |  |
| **51^st^-75^th^** | 19.7 | 20.7 | 22.4 |  |
| **76^th^–100^th^** | 14.2 | 16.2 | 19.8 |  |
| **Comorbidities, %** |  |  |  |  |
| **Dyslipidaemia** | 15.4 | 39.0 | 47.5 | <0.001 |
| **Thrombocytopenia** | 0.6 | 3.5 | 6.3 | <0.001 |
| **Smoking** | 12.7 | 14.1 | 7.9 | <0.001 |
| **Previous AMI** | 2.2 | 6.9 | 7.6 | <0.001 |
| **Previous PCI** | 1.6 | 5.9 | 6.1 | <0.001 |
| **Previous CABG** | 1.5 | 5.7 | 6.1 | <0.001 |
| **Anemias** | 4.8 | 28.7 | 40.3 | <0.001 |
| **Valvular disease** | 0.4 | 3.3 | 6.5 | <0.001 |
| **Peripheral vascular disorders** | 1.4 | 8.5 | 12.2 | <0.001 |
| **Coagulopathy** | 0.7 | 4.3 | 7.7 | <0.001 |
| **Diabetes Mellitus** | 63.3 | 63.6 | 59.4 | <0.001 |
| **Liver disease** | 1.4 | 4.2 | 4.0 | <0.001 |
| **Chronic renal failure** | 7.2 | 35.9 | 47.1 | <0.001 |
| **Chronic pulmonary disease** | 5.1 | 14.8 | 19.0 | <0.001 |
| **Cancer** | 2.0 | 5.8 | 6.5 | <0.001 |
| **Hospital Region, %** |  |  |  | <0.001 |
| **Northeast** | 16.7 | 16.1 | 18.5 |  |
| **Midwest** | 20.6 | 21.7 | 24.6 |  |
| **South** | 43.7 | 43.6 | 39.5 |  |
| **West** | 19.0 | 18.7 | 17.3 |  |
| **Location/teaching status of hospital, %** |  |  |  | <0.001 |
| **Rural** | 26.4 | 25.3 | 23.8 |  |
| **Urban non-teaching** | 55.8 | 61.3 | 64.8 |  |
| **Urban teaching** | 17.8 | 13.4 | 11.4 |  |
| **Length of stay (days), median (IQR)** | 2 (1, 4) | 3 (2, 6) | 6 (3, 10) | <0.001 |
| **Total ED and in-hospital charges (USD), median (IQR)** | 19,850 (12,422, 32,806) | 27,304 (16,138, 48,827) | 38,978 (21,976, 74,543) | <0.001 |
| **All-cause mortality, %** |  |  |  |  |
| **ED all-cause mortality** | <0.01 | <0.01 | <0.01 | <0.001 |
| **In-hospital all-cause mortality** | <0.01 | 0.7 | 3.3 | <0.001 |
| **Discharge disposition, %** |  |  |  | <0.001 |
| **Home** | 78.7 | 28.8 | 4.7 |  |
| **Transfer to short-term hospital** | 2.2 | 2.0 | 0.7 |  |
| **Other transfer** | 1.2 | 1.9 | 2.3 |  |
| **Home health care** | 0.3 | 1.1 | 1.3 |  |
| **Discharge against medical advice** | 1.8 | 0.7 | 0.1 |  |
| **Admitted as inpatient** | 15.7 | 65.6 | 91.0 |  |

**Abbreviations:** AMI – Acute Myocardial Infarction; CABG – Coronary Artery Bypass Graft; ED – Emergency Department; HFRS – Hospital Frailty Risk Score; IQR – Interquartile Range; PCI – Percutaneous Coronary Intervention; USD – United States Dollar.

**Supplementary Table 6.** Characteristics of patients presenting with neurological diseases.

| **Characteristics** | **Hospital Frailty Risk Score** | | | ***P*-value** |
| --- | --- | --- | --- | --- |
|  | **Low <5**  **(83.4%)** | **Intermediate 5-15**  **(15.3%)** | **High >15**  **(1.3%)** |  |
| **Number of weighted discharges** | 8,275,701 | 1,517,808 | 128,365 |  |
| **Age (years), median (IQR)** | 54 (38, 68) | 68 (55, 79) | 76 (65, 84) | <0.001 |
| **Female sex, %** | 63.3 | 54.7 | 56.5 | <0.001 |
| **Weekend admission, %** | 27.0 | 25.9 | 25.3 | <0.001 |
| **Primary expected payer, %** |  |  |  | <0.001 |
| **Medicare** | 24.5 | 62.7 | 79.8 |  |
| **Medicaid** | 29.4 | 14.9 | 8.2 |  |
| **Private Insurance** | 30.2 | 15.9 | 9.1 |  |
| **Self-pay** | 11.8 | 4.1 | 1.3 |  |
| **No charge** | 0.4 | 0.3 | 0.1 |  |
| **Other** | 3.6 | 2.2 | 1.5 |  |
| **Median Household Income (percentile), %** |  |  |  | <0.001 |
| **0-25^th^** | 36.0 | 32.8 | 31.3 |  |
| **26^th^–50^th^** | 28.3 | 26.5 | 28.0 |  |
| **51^st^-75^th^** | 20.7 | 22.0 | 21.0 |  |
| **76^th^–100^th^** | 15.0 | 18.7 | 15.7 |  |
| **Comorbidities, %** |  |  |  |  |
| **Dyslipidaemia** | 8.8 | 38.3 | 47.7 | <0.001 |
| **Thrombocytopenia** | 0.3 | 3.7 | 6.7 | <0.001 |
| **Smoking** | 14.4 | 12.9 | 8.0 | <0.001 |
| **Previous AMI** | 1.2 | 5.9 | 6.8 | <0.001 |
| **Previous PCI** | 0.8 | 5.2 | 6.0 | <0.001 |
| **Previous CABG** | 0.7 | 4.6 | 5.4 | <0.001 |
| **Anemias** | 1.8 | 16.6 | 30.1 | <0.001 |
| **Valvular disease** | 0.5 | 4.1 | 6.6 | <0.001 |
| **Peripheral vascular disorders** | 0.1 | 1.4 | 2.6 | <0.001 |
| **Coagulopathy** | 0.6 | 4.7 | 8.3 | <0.001 |
| **Diabetes Mellitus** | 10.5 | 29.6 | 35.0 | <0.001 |
| **Liver disease** | 0.5 | 2.9 | 3.3 | <0.001 |
| **Chronic renal failure** | 1.4 | 17.9 | 33.5 | <0.001 |
| **Chronic pulmonary disease** | 3.5 | 14.3 | 17.9 | <0.001 |
| **Cancer** | 1.6 | 6.3 | 5.7 | <0.001 |
| **Hospital Region, %** |  |  |  | <0.001 |
| **Northeast** | 16.0 | 16.6 | 18.3 |  |
| **Midwest** | 23.6 | 22.6 | 24.7 |  |
| **South** | 40.2 | 41.5 | 39.0 |  |
| **West** | 20.2 | 19.3 | 18.0 |  |
| **Location/teaching status of hospital, %** |  |  |  | <0.001 |
| **Rural** | 27.2 | 24.5 | 21.8 |  |
| **Urban non-teaching** | 54.4 | 63.9 | 69.8 |  |
| **Urban teaching** | 18.4 | 11.6 | 8.5 |  |
| **Length of stay (days), median (IQR)** | 2 (1, 4) | 3 (2, 6) | 6 (3, 11) | <0.001 |
| **Total ED and in-hospital charges (USD), median (IQR)** | 25,665 (15,842, 41,987) | 31,170 (18,921, 53,533) | 42,809 (24,588, 80,478) | <0.001 |
| **All-cause mortality, %** |  |  |  |  |
| **ED all-cause mortality** | <0.1 | <0.1 | <0.1 | <0.001 |
| **In-hospital all-cause mortality** | <0.1 | 1.0 | 3.5 | <0.001 |
| **Discharge disposition, %** |  |  |  | <0.001 |
| **Home** | 88.0 | 31.5 | 5.0 |  |
| **Transfer to short-term hospital** | 1.6 | 1.9 | 0.4 |  |
| **Other transfer** | 0.9 | 3.6 | 3.1 |  |
| **Home health care** | 0.3 | 1.8 | 1.5 |  |
| **Discharge against medical advice** | 1.5 | 0.7 | 0.1 |  |
| **Admitted as inpatient** | 7.8 | 60.4 | 89.8 |  |

**Abbreviations:** AMI – Acute Myocardial Infarction; CABG – Coronary Artery Bypass Graft; ED – Emergency Department; HFRS – Hospital Frailty Risk Score; IQR – Interquartile Range; PCI – Percutaneous Coronary Intervention; USD – United States Dollar.

**Supplementary Table 7.** Characteristics of patients presenting with cardiovascular diseases.

| **Characteristics** | **Hospital Frailty Risk Score** | | | ***P*-value** |
| --- | --- | --- | --- | --- |
|  | **Low <5**  **(66.0%)** | **Intermediate 5-15**  **(31.1%)** | **High >15**  **(2.9%)** |  |
| **Number of weighted discharges** | 13,520,067 | 6,384,446 | 594,427 |  |
| **Age (years), median (IQR)** | 66 (55, 77) | 73 (62, 82) | 77 (67, 86) | <0.001 |
| **Female sex, %** | 47.9 | 49.5 | 55.4 | <0.001 |
| **Weekend admission, %** | 24.4 | 24.8 | 25.9 | <0.001 |
| **Primary expected payer, %** |  |  |  | <0.001 |
| **Medicare** | 22.4 | 61.2 | 81.1 |  |
| **Medicaid** | 27.5 | 14.2 | 7.7 |  |
| **Private Insurance** | 30.4 | 16.9 | 8.5 |  |
| **Self-pay** | 14.6 | 4.9 | 1.3 |  |
| **No charge** | 0.5 | 0.3 | 0.1 |  |
| **Other** | 4.5 | 2.5 | 1.4 |  |
| **Median Household Income (percentile), %** |  |  |  | <0.001 |
| **0-25^th^** | 35.7 | 32.4 | 29.8 |  |
| **26^th^–50^th^** | 27.5 | 26.6 | 25.2 |  |
| **51^st^-75^th^** | 20.8 | 22.1 | 22.8 |  |
| **76^th^–100^th^** | 16.0 | 18.9 | 22.2 |  |
| **Comorbidities, %** |  |  |  |  |
| **Dyslipidaemia** | 29.5 | 53.9 | 55.2 | <0.001 |
| **Thrombocytopenia** | 1.3 | 5.6 | 6.8 | <0.001 |
| **Smoking** | 12.5 | 11.6 | 9.2 | <0.001 |
| **Previous AMI** | 7.1 | 13.1 | 9.6 | <0.001 |
| **Previous PCI** | 7.1 | 12.2 | 7.8 | <0.001 |
| **Previous CABG** | 5.3 | 11.1 | 7.7 | <0.001 |
| **Anemias** | 6.7 | 30.0 | 30.8 | <0.001 |
| **Valvular disease** | 5.4 | 16.2 | 14.6 | <0.001 |
| **Peripheral vascular disorders** | 1.2 | 4.6 | 4.8 | <0.001 |
| **Coagulopathy** | 2.0 | 7.5 | 9.5 | <0.001 |
| **Diabetes Mellitus** | 23.7 | 43.4 | 41.0 | <0.001 |
| **Liver disease** | 1.2 | 3.9 | 3.3 | <0.001 |
| **Chronic renal failure** | 10.1 | 44.0 | 43.2 | <0.001 |
| **Chronic pulmonary disease** | 10.5 | 26.4 | 21.6 | <0.001 |
| **Cancer** | 2.4 | 5.4 | 5.1 | <0.001 |
| **Hospital Region, %** |  |  |  | <0.001 |
| **Northeast** | 16.9 | 17.6 | 18.1 |  |
| **Midwest** | 22.0 | 22.9 | 25.1 |  |
| **South** | 42.9 | 41.9 | 39.8 |  |
| **West** | 18.3 | 17.6 | 17.0 |  |
| **Location/teaching status of hospital, %** |  |  |  | <0.001 |
| **Rural** | 26.9 | 24.8 | 20.9 |  |
| **Urban non-teaching** | 55.7 | 64.7 | 71.9 |  |
| **Urban teaching** | 17.4 | 10.5 | 7.2 |  |
| **Length of stay (days), median (IQR)** | 2 (2, 4) | 4 (2, 7) | 6 (4, 11) | <0.001 |
| **Total ED and in-hospital charges (USD), median (IQR)** | 31,171 (17,263, 59,618) | 38,281 (21,701, 72,277) | 58,356 (31,677, 115,054) | <0.001 |
| **All-cause mortality, %** |  |  |  | <0.001 |
| **ED all-cause mortality** | 3.1 | 0.4 | 0.1 |  |
| **In-hospital all-cause mortality** | 0.4 | 3.8 | 8.3 |  |
| **Discharge disposition, %** |  |  |  | <0.001 |
| **Home** | 53.2 | 10.4 | 1.2 |  |
| **Transfer to short-term hospital** | 7.3 | 2.4 | 0.4 |  |
| **Other transfer** | 1.5 | 1.2 | 0.6 |  |
| **Home health care** | 0.4 | 0.8 | 0.4 |  |
| **Discharge against medical advice** | 1.3 | 0.3 | <0.1 |  |
| **Admitted as inpatient** | 33.2 | 84.5 | 97.3 |  |

**Abbreviations:** AMI – Acute Myocardial Infarction; CABG – Coronary Artery Bypass Graft; ED – Emergency Department; HFRS – Hospital Frailty Risk Score; IQR – Interquartile Range; PCI – Percutaneous Coronary Intervention; USD – United States Dollar.

**Supplementary Table 8.** Characteristics of patients presenting with respiratory diseases.

| **Characteristics** | **Hospital Frailty Risk Score** | | | ***P*-value** |
| --- | --- | --- | --- | --- |
|  | **Low <5**  **(85.3%)** | **Intermediate 5-15**  **(13.8%)** | **High >15**  **(0.8%)** |  |
| **Number of weighted discharges** | 25,860,819 | 4,192,356 | 252,913 |  |
| **Age (years), median (IQR)** | 3 (2, 4) | 4 (3, 7) | 7 (4, 11) | <0.001 |
| **Female sex, %** | 60.7 | 55.4 | 55.0 | <0.001 |
| **Weekend admission, %** | 28.4 | 26.4 | 26.7 | <0.001 |
| **Primary expected payer, %** |  |  |  | <0.001 |
| **Medicare** | 24.8 | 73.1 | 84.7 |  |
| **Medicaid** | 28.8 | 11.0 | 6.4 |  |
| **Private Insurance** | 27.4 | 11.8 | 6.7 |  |
| **Self-pay** | 15.5 | 2.2 | 0.9 |  |
| **No charge** | 0.5 | 0.1 | <0.1 |  |
| **Other** | 3.0 | 1.8 | 1.3 |  |
| **Median Household Income (percentile), %** |  |  |  | <0.001 |
| **0-25^th^** | 39.1 | 33.3 | 30.3 |  |
| **26^th^–50^th^** | 28.4 | 27.1 | 25.5 |  |
| **51^st^-75^th^** | 19.5 | 21.9 | 22.6 |  |
| **76^th^–100^th^** | 13.1 | 17.8 | 21.7 |  |
| **Comorbidities, %** |  |  |  |  |
| **Dyslipidaemia** | 8.5 | 42.4 | 44.2 | <0.001 |
| **Thrombocytopenia** | 0.4 | 5.1 | 7.9 | <0.001 |
| **Smoking** | 17.1 | 16.1 | 6.9 | <0.001 |
| **Previous AMI** | 1.6 | 8.6 | 7.6 | <0.001 |
| **Previous PCI** | 1.2 | 7.8 | 5.7 | <0.001 |
| **Previous CABG** | 1.0 | 6.5 | 5.9 | <0.001 |
| **Anemias** | 2.0 | 26.7 | 40.9 | <0.001 |
| **Valvular disease** | 0.5 | 7.0 | 9.7 | <0.001 |
| **Peripheral vascular disorders** | 0.1 | 2.1 | 3.2 | <0.001 |
| **Coagulopathy** | 0.5 | 6.3 | 9.9 | <0.001 |
| **Diabetes Mellitus** | 10.7 | 35.4 | 37.5 | <0.001 |
| **Liver disease** | 0.5 | 3.5 | 3.5 | <0.001 |
| **Chronic renal failure** | 1.7 | 27.8 | 44.3 | <0.001 |
| **Chronic pulmonary disease** | 33.1 | 61.9 | 46.6 | <0.001 |
| **Cancer** | 1..2 | 8.5 | 6.9 | <0.001 |
| **Hospital Region, %** |  |  |  | <0.001 |
| **Northeast** | 17.0 | 17.9 | 18.5 |  |
| **Midwest** | 23.4 | 24.9 | 26.0 |  |
| **South** | 42.6 | 40.4 | 39.2 |  |
| **West** | 17.0 | 16.9 | 16.2 |  |
| **Location/teaching status of hospital, %** |  |  |  | <0.001 |
| **Rural** | 28.4 | 27.3 | 26.1 |  |
| **Urban non-teaching** | 52.4 | 57.6 | 61.8 |  |
| **Urban teaching** | 19.1 | 15.1 | 12.1 |  |
| **Length of stay (days), median (IQR)** | 3 (2, 4) | 4 (3, 7) | 7 (4, 11) | <0.001 |
| **Total ED and in-hospital charges (USD), median (IQR)** | 22,246 (13,932, 36,247) | 32,133 (19,025, 57,087) | 50,459 (27,615, 98,090) | <0.001 |
| **All-cause mortality, %** |  |  |  | <0.001 |
| **ED all-cause mortality** | <0.1 | 0.2 | 0.1 |  |
| **In-hospital all-cause mortality** | 0.1 | 3.9 | 9.8 |  |
| **Discharge disposition, %** |  |  |  | <0.001 |
| **Home** | 86.7 | 34.0 | 3.1 |  |
| **Transfer to short-term hospital** | 1.7 | 2.1 | 0.4 |  |
| **Other transfer** | 1.4 | 2.7 | 1.5 |  |
| **Home health care** | 0.2 | 0.9 | 0.7 |  |
| **Discharge against medical advice** | 1.9 | 0.6 | <0.1 |  |
| **Admitted as inpatient** | 8.0 | 59.6 | 94.2 |  |

**Abbreviations:** AMI – Acute Myocardial Infarction; CABG – Coronary Artery Bypass Graft; ED – Emergency Department; HFRS – Hospital Frailty Risk Score; IQR – Interquartile Range; PCI – Percutaneous Coronary Intervention; USD – United States Dollar.

**Supplementary Table 9.** Characteristics of patients presenting with gastrointestinal diseases.

| **Characteristics** | **Hospital Frailty Risk Score** | | | ***P*-value** |
| --- | --- | --- | --- | --- |
|  | **Low <5**  **(86.2%)** | **Intermediate 5-15**  **(13.3%)** | **High >15**  **(0.5%)** |  |
| **Number of weighted discharges** | 23,296,469 | 3,593,199 | 146,068 |  |
| **Age (years), median (IQR)** | 56 (42, 69) | 68 (56, 79) | 76 (65, 85) | <0.001 |
| **Female sex, %** | 54.7 | 55.5 | 59.3 | <0.001 |
| **Weekend admission, %** | 27.7 | 26.0 | 25.9 | <0.001 |
| **Primary expected payer, %** |  |  |  | <0.001 |
| **Medicare** | 24.0 | 62.4 | 79.2 |  |
| **Medicaid** | 26.0 | 13.9 | 9.0 |  |
| **Private Insurance** | 31.0 | 17.4 | 9.0 |  |
| **Self-pay** | 15.4 | 4.1 | 1.4 |  |
| **No charge** | 0.6 | 0.3 | 0.1 |  |
| **Other** | 2.9 | 1.9 | 1.3 |  |
| **Median Household Income (percentile), %** |  |  |  | <0.001 |
| **0-25^th^** | 35.4 | 31.8 | 30.4 |  |
| **26^th^–50^th^** | 28.0 | 26.4 | 25.4 |  |
| **51^st^-75^th^** | 20.9 | 22.5 | 23.0 |  |
| **76^th^–100^th^** | 15.8 | 19.3 | 21.2 |  |
| **Comorbidities, %** |  |  |  |  |
| **Dyslipidaemia** | 10.1 | 37.9 | 42.3 | <0.001 |
| **Thrombocytopenia** | 1.0 | 9.0 | 11.8 | <0.001 |
| **Smoking** | 15.0 | 13.7 | 8.0 | <0.001 |
| **Previous AMI** | 1.5 | 6.9 | 6.9 | <0.001 |
| **Previous PCI** | 1.3 | 6.6 | 5.4 | <0.001 |
| **Previous CABG** | 1.0 | 5.6 | 4.9 | <0.001 |
| **Anemias** | 4.2 | 30.7 | 42.7 | <0.001 |
| **Valvular disease** | 0.5 | 4.8 | 7.5 | <0.001 |
| **Peripheral vascular disorders** | 0.1 | 1.9 | 3.0 | <0.001 |
| **Coagulopathy** | 1.4 | 12.3 | 16.6 | <0.001 |
| **Diabetes Mellitus** | 11.1 | 32.1 | 35.6 | <0.001 |
| **Liver disease** | 3.7 | 15.6 | 14.5 | <0.001 |
| **Chronic renal failure** | 2.0 | 26.4 | 45.0 | <0.001 |
| **Chronic pulmonary disease** | 3.7 | 18.4 | 22.9 | <0.001 |
| **Cancer** | 1.5 | 7.9 | 7.6 | <0.001 |
| **Hospital Region, %** |  |  |  | <0.001 |
| **Northeast** | 18.1 | 17.1 | 18.1 |  |
| **Midwest** | 21.9 | 22.7 | 25.6 |  |
| **South** | 41.2 | 41.1 | 39.2 |  |
| **West** | 18.9 | 19.1 | 17.1 |  |
| **Location/teaching status of hospital, %** |  |  |  | <0.001 |
| **Rural** | 27.8 | 26.2 | 24.9 |  |
| **Urban non-teaching** | 55.2 | 63.0 | 66.1 |  |
| **Urban teaching** | 17.0 | 10.9 | 9.0 |  |
| **Length of stay (days), median (IQR)** | 3 (2, 4) | 4 (3, 7) | 7 (4, 13) | <0.001 |
| **Total ED and in-hospital charges (USD), median (IQR)** | 27,469 (16,731, 33,783) | 35,671 (20,834, 63,685) | 56,364 (30,377, 111,373) |  |
| **All-cause mortality, %** |  |  |  | <0.001 |
| **ED all-cause mortality** | <0.1 | <0.1 | 0.1 |  |
| **In-hospital all-cause mortality** | <0.1 | 2.2 | 7.4 |  |
| **Discharge disposition, %** |  |  |  | <0.001 |
| **Home** | 77.7 | 16.0 | 1.5 |  |
| **Transfer to short-term hospital** | 2.1 | 1.2 | 0.2 |  |
| **Other transfer** | 0.7 | 1.1 | 0.7 |  |
| **Home health care** | 0.1 | 0.5 | 0.4 |  |
| **Discharge against medical advice** | 0.9 | 0.2 | <0.1 |  |
| **Admitted as inpatient** | 18.5 | 80.9 | 97.2 |  |

**Abbreviations:** AMI – Acute Myocardial Infarction; CABG – Coronary Artery Bypass Graft; ED – Emergency Department; HFRS – Hospital Frailty Risk Score; IQR – Interquartile Range; PCI – Percutaneous Coronary Intervention; USD – United States Dollar.

**Supplementary Table 10.** Characteristics of patients presenting with musculoskeletal diseases.

| **Characteristics** | **Hospital Frailty Risk Score** | | | ***P*-value** |
| --- | --- | --- | --- | --- |
|  | **Low <5**  **(93.6%)** | **Intermediate 5-15**  **(6.3%)** | **High >15**  **(0.2%)** |  |
| **Number of weighted discharges** | 26,698,682 | 1,786,281 | 52,895 |  |
| **Age (years), median (IQR)** | 57 (42, 71) | 70 (57, 82) | 79 (69, 87) | <0.001 |
| **Female sex, %** | 56.2 | 61.2 | 62.1 | <0.001 |
| **Weekend admission, %** | 27.3 | 26.8 | 25.9 | <0.001 |
| **Primary expected payer, %** |  |  |  | <0.001 |
| **Medicare** | 24.7 | 61.8 | 83.9 |  |
| **Medicaid** | 26.4 | 14.6 | 6.0 |  |
| **Private Insurance** | 29.1 | 15.7 | 7.8 |  |
| **Self-pay** | 14.2 | 5.1 | 1.1 |  |
| **No charge** | 0.5 | 0.2 | 0.1 |  |
| **Other** | 5.0 | 2.6 | 1.1 |  |
| **Median Household Income (percentile), %** |  |  |  | <0.001 |
| **0-25^th^** | 37.8 | 34.1 | 27.3 |  |
| **26^th^–50^th^** | 27.3 | 26.2 | 24.2 |  |
| **51^st^-75^th^** | 20.1 | 21.8 | 24.1 |  |
| **76^th^–100^th^** | 14.8 | 17.9 | 24.3 |  |
| **Comorbidities, %** |  |  |  |  |
| **Dyslipidaemia** | 7.3 | 32.9 | 45.5 | <0.001 |
| **Thrombocytopenia** | 0.1 | 2.0 | 6.1 | <0.001 |
| **Smoking** | 13.2 | 11.9 | 7.1 | <0.001 |
| **Previous AMI** | 1.1 | 6.3 | 6.6 | <0.001 |
| **Previous PCI** | 0.7 | 4.9 | 5.4 | <0.001 |
| **Previous CABG** | 0.6 | 4.2 | 5.3 | <0.001 |
| **Anemias** | 1.0 | 13.8 | 34.5 | <0.001 |
| **Valvular disease** | 0.2 | 2.8 | 7.6 | <0.001 |
| **Peripheral vascular disorders** | 0.1 | 1.7 | 4.0 | <0.001 |
| **Coagulopathy** | 0.3 | 3.1 | 8.4 | <0.001 |
| **Diabetes Mellitus** | 10.6 | 30.6 | 32.6 | <0.001 |
| **Liver disease** | 0.3 | 2.3 | 3.4 | <0.001 |
| **Chronic renal failure** | 1.3 | 19.5 | 37.1 | <0.001 |
| **Chronic pulmonary disease** | 3.0 | 15.2 | 20.0 | <0.001 |
| **Cancer** | 0.6 | 3.9 | 6.6 | <0.001 |
| **Hospital Region, %** |  |  |  | <0.001 |
| **Northeast** | 19.9 | 16.2 | 19.7 |  |
| **Midwest** | 22.8 | 25.9 | 29.6 |  |
| **South** | 40.2 | 40.3 | 34.2 |  |
| **West** | 17.0 | 17.5 | 17.5 |  |
| **Location/teaching status of hospital, %** |  |  |  | <0.001 |
| **Rural** | 25.9 | 23.9 | 22.9 |  |
| **Urban non-teaching** | 58.5 | 63.4 | 67.5 |  |
| **Urban teaching** | 15.6 | 12.7 | 9.7 |  |
| **Length of stay (days), median (IQR)** | 3 (2, 5) | 5 (3, 7) | 6 (4, 11) | <0.001 |
| **Total ED and in-hospital charges (USD), median (IQR)** | 29,254 (16,832, 51,485) | 38,341 (21,641, 69,613) | 48,877 (26,130, 93,649) | <0.001 |
| **All-cause mortality, %** |  |  |  |  |
| **ED all-cause mortality** | <0.1 | <0.1 | <0.1 | <0.001 |
| **In-hospital all-cause mortality** | <0.1 | 0.3 | 2.6 | <0.001 |
| **Discharge disposition, %** |  |  |  | <0.001 |
| **Home** | 95.0 | 59.1 | 6.3 |  |
| **Transfer to short-term hospital** | 0.5 | 1.0 | 0.4 |  |
| **Other transfer** | 0..5 | 3.8 | 4.9 |  |
| **Home health care** | 0.1 | 1.3 | 1.6 |  |
| **Discharge against medical advice** | 1.7 | 0.8 | 0.1 |  |
| **Admitted as inpatient** | 2.2 | 34.0 | 86.6 |  |

**Abbreviations:** AMI – Acute Myocardial Infarction; CABG – Coronary Artery Bypass Graft; ED – Emergency Department; HFRS – Hospital Frailty Risk Score; IQR – Interquartile Range; PCI – Percutaneous Coronary Intervention; USD – United States Dollar.

**Supplementary Table 11.** Characteristics of patients presenting with psychiatric diseases.

| **Characteristics** | **Hospital Frailty Risk Score** | | | ***P*-value** |
| --- | --- | --- | --- | --- |
|  | **Low <5**  **(91.8%)** | **Intermediate 5-15**  **(8.0%)** | **High >15**  **(0.2%)** |  |
| **Number of weighted discharges** | 14,439,497 | 1,251,004 | 34,933 |  |
| **Age (years), median (IQR)** | 40 (29, 52) | 54 (41, 65) | 70 (59, 82) | <0.001 |
| **Female sex, %** | 43.0 | 45.7 | 47.5 | <0.001 |
| **Weekend admission, %** | 27.7 | 25.5 | 23.2 | <0.001 |
| **Primary expected payer, %** |  |  |  | <0.001 |
| **Medicare** | 18.5 | 40.5 | 66.6 |  |
| **Medicaid** | 36.9 | 29.0 | 15.4 |  |
| **Private Insurance** | 22.5 | 16.3 | 11.5 |  |
| **Self-pay** | 18.0 | 10.5 | 3.8 |  |
| **No charge** | 0.7 | 0.8 | 0.4 |  |
| **Other** | 3.3 | 3.0 | 2.2 |  |
| **Median Household Income (percentile), %** |  |  |  | <0.001 |
| **0-25^th^** | 34.1 | 33.8 | 29.8 |  |
| **26^th^–50^th^** | 26.0 | 25.7 | 25.2 |  |
| **51^st^-75^th^** | 21.2 | 21.9 | 22.5 |  |
| **76^th^–100^th^** | 18.7 | 18.6 | 22.5 |  |
| **Comorbidities, %** |  |  |  |  |
| **Dyslipidaemia** | 4.8 | 22.0 | 38.0 | <0.001 |
| **Thrombocytopenia** | 0.5 | 7.2 | 13.0 | <0.001 |
| **Smoking** | 22.5 | 29.7 | 18.3 | <0.001 |
| **Previous AMI** | 0.8 | 3.7 | 5.5 | <0.001 |
| **Previous PCI** | 0.4 | 2.3 | 3.8 | <0.001 |
| **Previous CABG** | 0.3 | 1.9 | 3.6 | <0.001 |
| **Anemias** | 1.4 | 13.1 | 29.8 | <0.001 |
| **Valvular disease** | 0.1 | 1.3 | 4.2 | <0.001 |
| **Peripheral vascular disorders** | <0.1 | 0.5 | 1.5 | <0.001 |
| **Coagulopathy** | 0.6 | 7.8 | 14.3 | <0.001 |
| **Diabetes Mellitus** | 6.4 | 17.8 | 24.4 | <0.001 |
| **Liver disease** | 1.0 | 6.3 | 8.3 | <0.001 |
| **Chronic renal failure** | 0.6 | 8.0 | 24.6 | <0.001 |
| **Chronic pulmonary disease** | 3.4 | 13.3 | 18.7 | <0.001 |
| **Cancer** | 0.3 | 1.3 | 2.5 | <0.001 |
| **Hospital Region, %** |  |  |  | <0.001 |
| **Northeast** | 27.0 | 20.5 | 21.9 |  |
| **Midwest** | 21.2 | 25.1 | 25.9 |  |
| **South** | 31.0 | 36.5 | 34.3 |  |
| **West** | 20.8 | 17.9 | 18.0 |  |
| **Location/teaching status of hospital, %** |  |  |  | <0.001 |
| **Rural** | 22.7 | 21.2 | 20.6 |  |
| **Urban non-teaching** | 65.4 | 67.6 | 68.6 |  |
| **Urban teaching** | 11.9 | 11.1 | 10.8 |  |
| **Length of stay (days), median (IQR)** | 4 (2, 7) | 5 (3, 8) | 8 (4, 15) | <0.001 |
| **Total ED and in-hospital charges (USD), median (IQR)** | 14,960 (9,095, 25,562) | 23,272 (13,773, 40,641) | 40,924 (23,449, 78,377) | <0.001 |
| **All-cause mortality, %** |  |  |  |  |
| **ED all-cause mortality** | <0.1 | <0.1 | <0.1 | <0.001 |
| **In-hospital all-cause mortality** | <0.1 | 0.2 | 1.5 | <0.001 |
| **Discharge disposition, %** |  |  |  | <0.001 |
| **Home** | 71.3 | 28.1 | 4.5 |  |
| **Transfer to short-term hospital** | 1.9 | 1.2 | 0.4 |  |
| **Other transfer** | 8.3 | 5.7 | 2.6 |  |
| **Home health care** | 0.1 | 0.4 | 0.8 |  |
| **Discharge against medical advice** | 2.3 | 1.0 | 0.1 |  |
| **Admitted as inpatient** | 16.0 | 63.6 | 91.5 |  |

**Abbreviations:** AMI – Acute Myocardial Infarction; CABG – Coronary Artery Bypass Graft; ED – Emergency Department; HFRS – Hospital Frailty Risk Score; IQR – Interquartile Range; PCI – Percutaneous Coronary Intervention; USD – United States Dollar.

**Supplementary Table 12.** Adjusted odds of ED mortality by admission cause stratified by HFRS modelled as a continuous variable*.

| **Admission Diagnosis** | | **Hospital Frailty Risk Score** | |
| --- | --- | --- | --- |
|  |  | **aOR** | ***P-*value** |
| **Infectious Diseases (N =11,201,562)** | **Hospitalisation** | 1.98 [1.98-1.98] | <0.001 |
|  | **ED mortality** | 0.95 [0.95-0.95] | <0.001 |
|  | **Overall mortality** | 1.12 [1.12-1.12] | <0.001 |
| **Neoplasm (N =2,110,162)** | **Hospitalisation** | 1.61 [1.61-1.61] | <0.001 |
|  | **ED mortality** | 0.91 [0.90-0.92] | <0.001 |
|  | **Overall mortality** | 1.20 [1.20-1.20] | <0.001 |
| **Haematological Diseases (N =2,296,711)** | **Hospitalisation** | 1.48 [1.48-1.48] | <0.001 |
|  | **ED mortality** | 1.04 [1.02-1.06] | <0.001 |
|  | **Overall mortality** | 1.26 [1.25-1.26] | <0.001 |
| **Endocrine Diseases (N =7,862,684)** | **Hospitalisation** | 1.48 [1.48-1.48] | <0.001 |
|  | **ED mortality** | 1.00 [0.99-1.01] | 0.609 |
|  | **Overall mortality** | 1.22 [1.22-1.22] | <0.001 |
| **Neurological Diseases (N =9,921,873)** | **Hospitalisation** | 1.47 [1.47-1.47] | <0.001 |
|  | **ED mortality** | 1.02 [1.01-1.04] | <0.001 |
|  | **Overall mortality** | 1.22 [1.21-1.22] | <0.001 |
| **Cardiovascular Diseases**  **(N =20,498,940)** | **Hospitalisation** | 1.44 [1.44-1.44] | <0.001 |
|  | **ED mortality** | 0.73 [0.73-0.73] | <0.001 |
|  | **Overall mortality** | 1.05 [1.05-1.05] | <0.001 |
| **Respiratory Diseases (N =30,306,088)** | **Hospitalisation** | 1.94 [1.94-1.94] | <0.001 |
|  | **ED mortality** | 1.06 [1.05-1.06] | <0.001 |
|  | **Overall mortality** | 1.25 [1.24-1.25] | <0.001 |
| **Gastrointestinal Diseases**  **(N=27,035,737)** | **Hospitalisation** | 1.55 [1.55-1.55] | <0.001 |
|  | **ED mortality** | 1.06 [1.05-1.07] | <0.001 |
|  | **Overall mortality** | 1.28 [1.28-1.29] | <0.001 |
| **Musculoskeletal Diseases**  **(N=28,537,858)** | **Hospitalisation** | 1.55 [1.55-1.55] | <0.001 |
|  | **ED mortality** | 1.14 [1.12-1.16] | <0.001 |
|  | **Overall mortality** | 1.34 [1.33-1.34] | <0.001 |
| **Psychiatric Diseases (N =15,725,433)** | **Hospitalisation** | 1.52 [1.52-1.52] | <0.001 |
|  | **ED mortality** | 1.09 [1.07-1.10] | <0.001 |
|  | **Overall mortality** | 1.28 [1.27-1.29] | <0.001 |

*per 1-unit increase of HFRS.

**Multivariable logistic regression model** adjusted for: age, sex, weekend admission, primary expected payer, median household income, region and teaching status of the hospital, dyslipidemia, smoking, previous AMI, coagulopathies, chronic renal disease and valvular heart diseases.

**Abbreviations:** aOR – adjusted Odds Ratio; CI – Confidence Interval; ED – Emergency Department; HFRS – Hospital Frailty Risk Score.

**Supplementary Table 13.** Most common diagnoses from each cause of ED encounter stratified by HFRS*.

| **Admission diagnosis­** | **Hospital Frailty Risk Score** | | | | | |
| --- | --- | --- | --- | --- | --- | --- |
|  | **Low <5**  **(80.9%)** | | **Intermediate 5-15**  **(17.5%)** | | **High >15**  **(1.6%)** | |
|  | **Diagnosis** | **Prevalence within overall low risk cohort** | **Diagnosis** | **Prevalence within overall intermediate risk cohort** | **Diagnosis** | **Prevalence within overall high risk cohort** |
| **Infectious Diseases (N =11,201,562)** | **Viral infection (unspecified)** | 1.2 | **Sepsis (unspecified organism)** | 11.3 | **Sepsis (unspecified organism)** | 31.2 |
|  | **Sepsis (unspecified organism)** | 0.4 | **Sepsis due to Escherichia coli [E. coli]** | 0.9 | **Sepsis due to Escherichia coli [E. coli]** | 3.2 |
|  | **Zoster without complications** | 0.4 | **Enterocolitis due to Clostridium difficile** | 0.4 | **Other specified sepsis** | 1.2 |
| **Neoplasms (N =2,110,162)** | **Leiomyoma of uterus, unspecified** | 0.1 | **Secondary malignant neoplasm of brain** | 0.2 | **Secondary malignant neoplasm of brain** | 0.1 |
|  | **Malignant neoplasm of unspecified part of unspecified bronchus or lung** | <0.1 | **Secondary malignant neoplasm of bone** | 0.1 | **Secondary malignant neoplasm of bone** | 0.1 |
|  | **Secondary malignant neoplasm of brain** | <0.1 | **Malignant neoplasm of unspecified part of unspecified bronchus or lung** | 0.1 | **Malignant neoplasm of bladder, unspecified** | 0.1 |
| **Haematological Diseases (N =2,296,711)** | **Hb-SS disease with crisis, unspecified** | 0.4 | **Anaemia, unspecified** | 0.3 | **Acute posthemorrhagic anaemia** | 0.2 |
|  | **Anaemia, unspecified** | 0.3 | **Acute posthemorrhagic anaemia** | 0.3 | **Anaemia, unspecified** | 0.1 |
|  | **Iron deficiency anaemia, unspecified** | 0.1 | **Iron deficiency anaemia, unspecified** | 0.2 | **Iron deficiency anaemia, unspecified** | 0.1 |
| **Endocrine Diseases (N =7,862,684)** | **Type 2 diabetes mellitus with hyperglycemia** | 0.9 | **Dehydration** | 1.8 | **Dehydration** | 1.0 |
|  | **Dehydration** | 0.8 | **Hypo-osmolality and hyponatremia** | 0.9 | **Hypo-osmolality and hyponatremia** | 0.7 |
|  | **Type 2 diabetes mellitus with hypoglycemia without coma** | 0.3 | **Hyperkalemia** | 0.7 | **Hyperosmolality and hypernatremia** | 0.4 |
| **Neurological Diseases (N =9,921,873)** | **Migraine, unspecified, not intractable, without status migrainosus** | 1.3 | **Transient cerebral ischemic attack, unspecified** | 1.0 | **Transient cerebral ischemic attack, unspecified** | 0.7 |
|  | **Other chronic pain** | 0.8 | **Epilepsy, unspecified, not intractable, without status epilepticus** | 0.8 | **Metabolic encephalopathy** | 0.6 |
|  | **Epilepsy, unspecified, not intractable, without status epilepticus** | 0.7 | **Toxic encephalopathy** | 0.3 | **Epilepsy, unspecified, not intractable, without status epilepticus** | 0.5 |
| **Cardiovascular Diseases**  **(N =20,498,940)** | **Essential (primary) hypertension** | 2.1 | **Hypertensive heart and chronic kidney disease with heart failure and stage 1 through stage 4 chronic kidney disease, or unspecified chronic kidney disease** | 2.9 | **Cerebral infarction, unspecified** | 4.2 |
|  | **Non-ST elevation (NSTEMI) myocardial infarction** | 0.6 | **Cerebral infarction, unspecified** | 1.9 | **Hypertensive heart and chronic kidney disease with heart failure and stage 1 through stage 4 chronic kidney disease, or unspecified chronic kidney disease** | 2.0 |
|  | **Unspecified atrial fibrillation** | 0.6 | **Hypertensive heart disease with heart failure** | 1.6 | **Non-ST elevation (NSTEMI) myocardial infarction** | 1.0 |
| **Respiratory Diseases (N =30,306,088)** | **Acute upper respiratory infection, unspecified** | 2.7 | **Pneumonia, unspecified organism** | 3.4 | **Pneumonitis due to inhalation of food and vomit** | 2.5 |
|  | **Acute bronchitis, unspecified** | 2.0 | **Chronic obstructive pulmonary disease with (acute) exacerbation** | 2.7 | **Pneumonia, unspecified organism** | 2.3 |
|  | **Acute pharyngitis, unspecified** | 1.9 | **Acute respiratory failure with hypoxia** | 1.2 | **Acute respiratory failure with hypoxia** | 1.0 |
| **Gastrointestinal Diseases**  **(N=27,035,737)** | **Noninfective gastroenteritis and colitis, unspecified** | 2.0 | **Gastrointestinal hemorrhage, unspecified** | 0.9 | **Gastrointestinal hemorrhage, unspecified** | 0.6 |
|  | **Constipation, unspecified** | 1.2 | **Noninfective gastroenteritis and colitis, unspecified** | 0.8 | **Melena** | 0.2 |
|  | **Periapical abscess without sinus** | 1.1 | **Constipation, unspecified** | 0.7 | **Chronic or unspecified duodenal ulcer with hemorrhage** | 0.2 |
| **Musculoskeletal Diseases**  **(N=28,537,858)** | **Low back pain** | 3.4 | **Low back pain** | 0.4 | **Rhabdomyolysis** | 0.3 |
|  | **Cervicalgia** | 1.1 | **Pain in right hip** | 0.3 | **Collapsed vertebra, not elsewhere classified, lumbar region, initial encounter for fracture** | 0.1 |
|  | **Dorsalgia, unspecified** | 0.9 | **Pain in right knee** | 0.3 | **Age-related osteoporosis with current pathological fracture, vertebra(e), initial encounter for fracture** | 0.1 |
| **Psychiatric Diseases (N =15,725,433)** | **Anxiety disorder, unspecified** | 1.5 | **Alcohol dependence with withdrawal, unspecified** | 0.6 | **Alcohol dependence with withdrawal, unspecified** | 0.2 |
|  | **Alcohol abuse with intoxication, unspecified** | 1.2 | **Alcohol abuse with intoxication, unspecified** | 0.4 | **Alcohol dependence with withdrawal delirium** | 0.2 |
|  | **Major depressive disorder, single episode, unspecified** | 0.9 | **Unspecified dementia without behavioral disturbance** | 0.3 | **Unspecified dementia without behavioral disturbance** | 0.2 |

**Abbreviations:** aOR – adjusted Odds Ratio; CI – Confidence Interval; ED – Emergency Department; HFRS – Hospital Frailty Risk Score.

**Supplementary Table 14.** Characteristics of patients over 65 presenting to the ED.

| **Characteristics** | **Hospital Frailty Risk Score** | | | ***P*-value** |
| --- | --- | --- | --- | --- |
|  | **Low <5**  **(59.0%)** | **Intermediate 5-15**  **(36.5%)** | **High >15**  **(4.5%)** |  |
| **Number of weighted discharges** | 24,856,421 | 15,374,744 | 1,881,824 |  |
| **Age (years), median (IQR)** | 76 (70, 82) | 78 (72, 85) | 81 (74, 87) | <0.001 |
| **Female sex, %** | 57.1 | 55.2 | 57.1 | <0.001 |
| **Weekend admission, %** | 27.4 | 25.5 | 26.0 | <0.001 |
| **Primary expected payer, %** |  |  |  | <0.001 |
| **Medicare** | 87.1 | 90.7 | 92.0 |  |
| **Medicaid** | 1.7 | 1.4 | 1.6 |  |
| **Private Insurance** | 8.2 | 6.1 | 4.8 |  |
| **Self-pay** | 1.4 | 0.6 | 0.5 |  |
| **No charge** | 0.1 | <0.1 | <0.1 |  |
| **Other** | 1.5 | 1.2 | 1.1 |  |
| **Median Household Income (percentile), %** |  |  |  | <0.001 |
| **0-25^th^** | 30.8 | 28.7 | 28.3 |  |
| **26^th^–50^th^** | 27.6 | 26.2 | 24.8 |  |
| **51^st^-75^th^** | 22.4 | 23.4 | 23.3 |  |
| **76^th^–100^th^** | 19.3 | 21.8 | 23.6 |  |
| **Comorbidities, %** |  |  |  |  |
| **Dyslipidaemia** | 27.1 | 50.0 | 48.7 | <0.001 |
| **Thrombocytopenia** | 1.3 | 6.1 | 9.0 | <0.001 |
| **Smoking** | 6.3 | 6.7 | 4.4 | <0.001 |
| **Previous AMI** | 4.9 | 9.8 | 7.7 | <0.001 |
| **Previous PCI** | 4.8 | 9.5 | 6.2 | <0.001 |
| **Previous CABG** | 4.5 | 9.4 | 6.7 | <0.001 |
| **Anemias** | 7.2 | 29.1 | 38.1 | <0.001 |
| **Valvular disease** | 3.1 | 10.5 | 10.7 | <0.001 |
| **Peripheral vascular disorders** | 0.8 | 3.7 | 4.5 | <0.001 |
| **Coagulopathy** | 1.9 | 7.9 | 11.6 | <0.001 |
| **Diabetes Mellitus** | 24.6 | 38.9 | 39.8 | <0.001 |
| **Liver disease** | 1.2 | 3.8 | 3.9 | <0.001 |
| **Chronic renal failure** | 7.3 | 34.5 | 44.8 | <0.001 |
| **Chronic pulmonary disease** | 17.7 | 31.4 | 26.7 | <0.001 |
| **Cancer** | 4.8 | 11.2 | 8.9 | <0.001 |
| **Hospital Region, %** |  |  |  | <0.001 |
| **Northeast** | 18.8 | 19.1 | 19.7 |  |
| **Midwest** | 22.3 | 23.2 | 23.8 |  |
| **South** | 39.5 | 38.8 | 37.3 |  |
| **West** | 19.3 | 18.9 | 19.2 |  |
| **Location/teaching status of hospital, %** |  |  |  | <0.001 |
| **Rural** | 28.3 | 27.0 | 25.4 |  |
| **Urban non-teaching** | 52.4 | 60.6 | 65.1 |  |
| **Urban teaching** | 19.3 | 12.4 | 9.5 |  |
| **Length of stay (days), median (IQR)** | 3 (2, 4) | 4 (3, 7) | 6 (4, 11) | <0.001 |
| **Total ED and in-hospital charges (USD), median (IQR)** | 28,078 (16,565, 49,596) | 36,448 (21,042, 66,486) | 53,056 (29,402, 100,438) | <0.001 |
| **All-cause mortality, %** |  |  |  | <0.001 |
| **ED all-cause mortality** | 1.0 | 0.2 | 0.1 |  |
| **Overall all-cause mortality** | 0.3 | 4.2 | 10.0 |  |
| **Discharge disposition, %** |  |  |  | <0.001 |
| **Home** | 66.9 | 14.4 | 1.3 |  |
| **Transfer to short-term hospital** | 4.5 | 1.9 | 0.3 |  |
| **Other transfer** | 2.2 | 2.0 | 0.9 |  |
| **Home health care** | 0.5 | 1.0 | 0.5 |  |
| **Discharge against medical advice** | 0.8 | 0.2 | <0.1 |  |
| **Admitted as inpatient** | 24.1 | 80.2 | 96.9 |  |

**Abbreviations:** AMI – Acute Myocardial Infarction; CABG – Coronary Artery Bypass Graft; ED – Emergency Department; HFRS – Hospital Frailty Risk Score; IQR – Interquartile Range; PCI – Percutaneous Coronary Intervention; USD – United States Dollar.

**Supplementary Table 15.** Adjusted odds of ED mortality by admission cause stratified by HFRS in patients aged over 65 only*.

| **Admission Diagnosis** | | **Hospital Frailty Risk Score** | | | |
| --- | --- | --- | --- | --- | --- |
|  |  | **Intermediate 5-15**  **(17.5%)** | | **High >15**  **(1.6%)** | |
|  |  | **aOR** | ***P-*value** | **aOR** | ***P-*value** |
| **Infectious Diseases (N =4,006,048)** | **Hospitalisation** | 36.67 [36.36-36.98] | <0.001 | 182.20 [178.00-185.51] | <0.001 |
|  | **ED mortality** | 0.83 [0.79-0.87] | <0.001 | 0.32 [0.29-0.34] | <0.001 |
|  | **Overall mortality** | 8.99 [8.74-9.19] | <0.001 | 12.05 [11.78-12.33] | <0.001 |
| **Neoplasms (N =956,629)** | **Hospitalisation** | 8.34 [8.22-8.45] | <0.001 | 47.63 [43.15-52.58] | <0.001 |
|  | **ED mortality** | 0.54 [0.49-0.59] | <0.001 | 0.09 [0.04-0.18] | <0.001 |
|  | **Overall mortality** | 4.29 [4.20-4.39] | <0.001 | 7.33 [7.05-7.63] | <0.001 |
| **Haematological Diseases (N =730,756)** | **Hospitalisation** | 6.28 [6.21-6.36] | <0.001 | 33.93 [31.58-36.47] | <0.001 |
|  | **ED mortality** | 1.21 [0.96-1.52] | 0.112 | 1.64 [0.86-3.11] | 0.130 |
|  | **Overall mortality** | 9.21 [8.56-9.92] | <0.001 | 26.52 [24.03-29.26] | <0.001 |
| **Endocrine Diseases (N =2,561,800)** | **Hospitalisation** | 8.23 [8.17-8.29] | <0.001 | 43.63 [42.68-44.60] | <0.001 |
|  | **ED mortality** | 1.71 [1.54-1.89] | <0.001 | 1.12 [0.84-1.49] | 0.447 |
|  | **Overall mortality** | 8.48 [8.01-8.97] | <0.001 | 27.00 [25.31-28.79] | <0.001 |
| **Neurological Diseases (N =2,022,161)** | **Hospitalisation** | 7.35 [7.30-7.41] | <0.001 | 34.49 [33.75-35.26] | <0.001 |
|  | **ED mortality** | 1.81 [1.58-2.08] | <0.001 | 0.60 [0.39-0.93] | 0.023 |
|  | **Overall mortality** | 7.45 [7.07-7.86] | <0.001 | 20.16 [18.97-21.43] | <0.001 |
| **Cardiovascular Diseases**  **(N =10,841,333)** | **Hospitalisation** | 7.22 [7.20-7.25] | <0.001 | 47.00 [46.16-47.85] | <0.001 |
|  | **ED mortality** | 0.20 [0.20-0.20] | <0.001 | 0.03 [0.02-0.03] | <0.001 |
|  | **Overall mortality** | 1.30 [1.29-1.31] | <0.001 | 2.46 [2.43-2.48] | <0.001 |
| **Respiratory Diseases (N =7,603,322)** | **Hospitalisation** | 17.45 [17.37-17.52] | <0.001 | 112.48 [109.28-115.77] | <0.001 |
|  | **ED mortality** | 1.61 [1.56-1.67] | <0.001 | 1.01 [0.89-1.14] | 0.901 |
|  | **Overall mortality** | 10.32 [10.16-10.48] | <0.001 | 22.24 [21.79-22.271] | <0.001 |
| **Gastrointestinal Diseases**  **(N=6,336,841)** | **Hospitalisation** | 7.64 [7.60-7.62] | <0.001 | 50.70 [48.94-52.52] | <0.001 |
|  | **ED mortality** | 1.55 [1.44-1.68] | <0.001 | 1.67 [1.32-2.10] | <0.001 |
|  | **Overall mortality** | 11.68 [1..40-11.96] | <0.001 | 31.45 [30.43-32.49] | <0.001 |
| **Musculoskeletal Diseases**  **(N=5,697,725)** | **Hospitalisation** | 9.55 [9.49-9.61] | <0.001 | 90.00 [87.46-92.62] | <0.001 |
|  | **ED mortality** | 3.36 [2.63-4.29] | <0.001 | n/a** | n/a** |
|  | **Overall mortality** | 24.91 -22.84-27.18] | <0.001 | 134.07 [120.94-148.62] | <0.001 |
| **Psychiatric Diseases (N =1,356,736)** | **Hospitalisation** | 7.35 [7.28-7.42] | <0.001 | 44.82 [42.72-47.02] | <0.001 |
|  | **ED mortality** | 3.15 [2.40-4.13] | <0.001 | 3.17 [1.59-6.32] | <0.001 |
|  | **Overall mortality** | 11.90 [10.42-13.59] | <0.001 | 50.16 [42.52-59.16] | <0.001 |

*Reference group is low HFRS score <5 for each admission diagnosis.

**Results could not be reported due to low number of cases.

**Multivariable logistic regression model** adjusted for: sex, weekend admission, primary expected payer, median household income, region and teaching status of the hospital, dyslipidemia, smoking, previous AMI, coagulopathies, chronic renal disease and valvular heart diseases.

**Abbreviations:** aOR – adjusted Odds Ratio; CI – Confidence Interval; ED – Emergency Department; HFRS – Hospital Frailty Risk Score.

**Supplementary Figure 1.** Flow diagram of the cohort selection process.


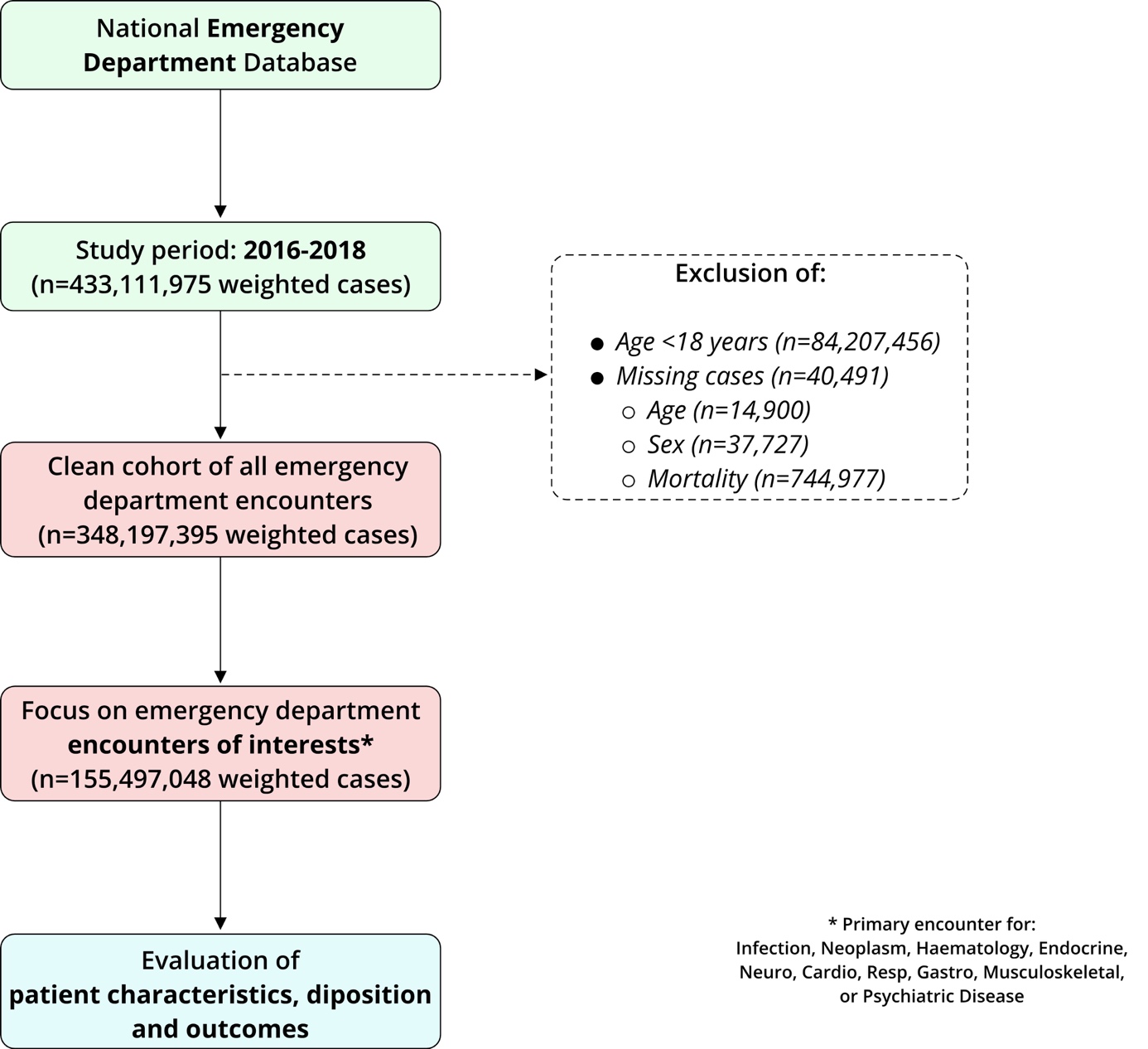


**Abbreviations:** ED – Emergency Department.

**Supplementary Figure 2**. Distribution of cause of admission within each HFRS category.

**
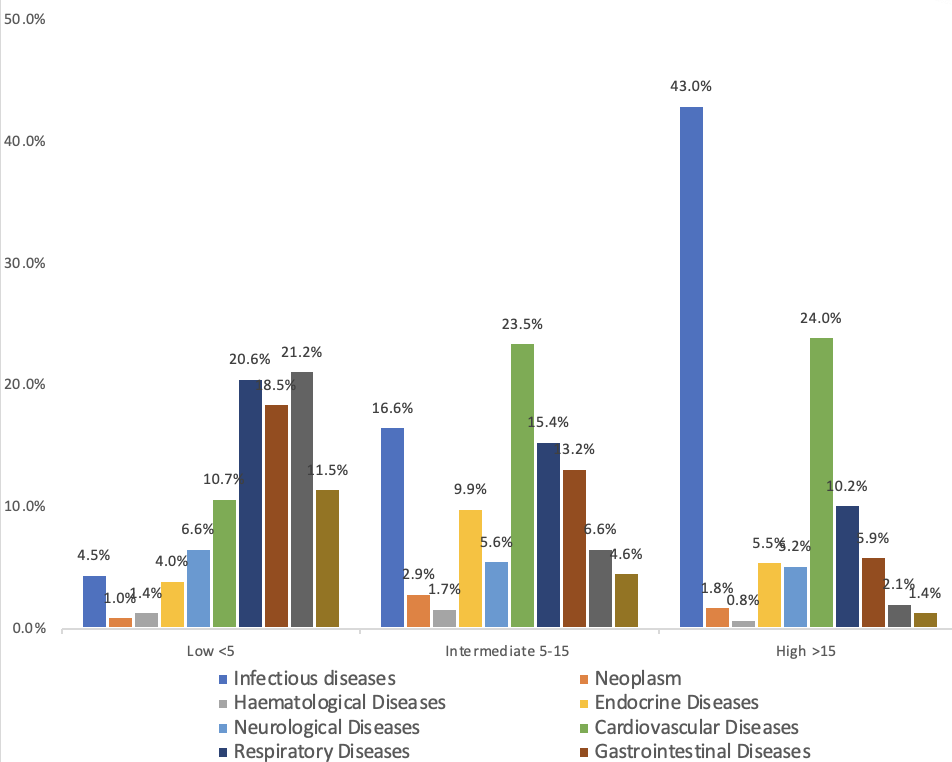
**

**Abbreviations:**  HFRS – Hospital Frailty Risk Score.

**Appendix 1.** Components of Hospital Frailty Risk Score with associated weighting.

| **Hospital Frailty Risk Score Variables** | **Codes** | **Source** | **Weighting** |
| --- | --- | --- | --- |
| **Dementia in Alzheimer's disease** | F00 | ICD-10 | 7.1 |
| **Hemiplegia** | G81 | ICD-10 | 4.4 |
| **Alzheimer's disease** | G30 | ICD-10 | 4 |
| **Sequelae of cerebrovascular disease** | I69 | ICD-10 | 3.7 |
| **Other symptoms and signs involving the nervous and musculoskeletal systems** | R29 | ICD-10 | 3.6 |
| **Other disorders of urinary system (includes urinary tract infection and urinary incontinence)** | N39 | ICD-10 | 3.2 |
| **Delirium, not induced by alcohol and other psychoactive substances** | F05 | ICD-10 | 3.2 |
| **Unspecified fall** | W19 | ICD-10 | 3.2 |
| **Superficial injury of head** | S00 | ICD-10 | 3.2 |
| **Unspecified haematuria** | R31 | ICD-10 | 3 |
| **Other bacterial agents as the cause of diseases classified to other chapters** | B96 | ICD-10 | 2.9 |
| **Other symptoms and signs involving cognitive functions and awareness** | R41 | ICD-10 | 2.7 |
| **Abnormalities of gait and mobility** | R26 | ICD-10 | 2.6 |
| **Other cerebrovascular diseases** | I67 | ICD-10 | 2.6 |
| **Convulsions, not elsewhere classified** | R56 | ICD-10 | 2.6 |
| **Somnolence, stupor and coma** | R40 | ICD-10 | 2.5 |
| **Complications of genitourinary prosthetic devices, implants and grafts** | T83 | ICD-10 | 2.4 |
| **Intracranial injury** | S06 | ICD-10 | 2.4 |
| **Fracture of shoulder and upper arm** | S42 | ICD-10 | 2.3 |
| **Other disorders of fluid, electrolyte and acid- base balance** | E87 | ICD-10 | 2.3 |
| **Other joint disorders, not elsewhere classified** | M25 | ICD-10 | 2.3 |
| **Volume depletion** | E86 | ICD-10 | 2.3 |
| **Senility** | R54 | ICD-10 | 2.2 |
| **Care involving use of rehabilitation procedures** | Z50 | ICD-10 | 2.1 |
| **Unspecified dementia** | F03 | ICD-10 | 2.1 |
| **Other fall on same level** | W18 | ICD-10 | 2.1 |
| **Problems related to medical facilities and other health care** | Z75 | ICD-10 | 2 |
| **Vascular dementia** | F01 | ICD-10 | 2 |
| **Superficial injury of lower leg** | S80 | ICD-10 | 2 |
| **Cellulitis** | L03 | ICD-10 | 2 |
| **Blindness and low vision** | H54 | ICD-10 | 1.9 |
| **Deficiency of other B group vitamins** | E53 | ICD-10 | 1.9 |
| **Problems related to social environment** | Z60 | ICD-10 | 1.8 |
| **Parkinson's disease** | G20 | ICD-10 | 1.8 |
| **Syncope and collapse** | R55 | ICD-10 | 1.8 |
| **Fracture of rib(s), sternum and thoracic spine** | S22 | ICD-10 | 1.8 |
| **Other functional intestinal disorders** | K59 | ICD-10 | 1.8 |
| **Acute renal failure** | N17 | ICD-10 | 1.8 |
| **Decubitus ulcer** | L89 | ICD-10 | 1.7 |
| **Carrier of infectious disease** | Z22 | ICD-10 | 1.7 |
| **Streptococcus and staphylococcus as the cause of diseases classified to other chapters** | B95 | ICD-10 | 1.7 |
| **Ulcer of lower limb, not elsewhere classified** | L97 | ICD-10 | 1.6 |
| **Other symptoms and signs involving general sensations and perceptions** | R44 | ICD-10 | 1.6 |
| **Duodenal ulcer** | K26 | ICD-10 | 1.6 |
| **Hypotension** | I95 | ICD-10 | 1.6 |
| **Unspecified renal failure** | N19 | ICD-10 | 1.6 |
| **Other septicaemia** | A41 | ICD-10 | 1.6 |
| **Personal history of other diseases and conditions** | Z87 | ICD-10 | 1.5 |
| **Respiratory failure, not elsewhere classified** | J96 | ICD-10 | 1.5 |
| **Exposure to unspecified factor** | X59 | ICD-10 | 1.5 |
| **Other arthrosis** | M19 | ICD-10 | 1.5 |
| **Epilepsy** | G40 | ICD-10 | 1.5 |
| **Osteoporosis without pathological fracture** | M81 | ICD-10 | 1.4 |
| **Fracture of femur** | S72 | ICD-10 | 1.4 |
| **Fracture of lumbar spine and pelvis** | S32 | ICD-10 | 1.4 |
| **Other disorders of pancreatic internal secretion** | E16 | ICD-10 | 1.4 |
| **Abnormal results of function studies** | R94 | ICD-10 | 1.4 |
| **Chronic renal failure** | N18 | ICD-10 | 1.4 |
| **Retention of urine** | R33 | ICD-10 | 1.3 |
| **Unknown and unspecified causes of morbidity** | R69 | ICD-10 | 1.3 |
| **Other disorders of kidney and ureter, not elsewhere classified** | N28 | ICD-10 | 1.3 |
| **Unspecified urinary incontinence** | R32 | ICD-10 | 1.2 |
| **Other degenerative diseases of nervous system, not elsewhere classified** | G31 | ICD-10 | 1.2 |
| **Nosocomial condition** | Y95 | ICD-10 | 1.2 |
| **Other and unspecified injuries of head** | S09 | ICD-10 | 1.2 |
| **Symptoms and signs involving emotional state** | R45 | ICD-10 | 1.2 |
| **Transient cerebral ischaemic attacks and related syndromes** | G45 | ICD-10 | 1.2 |
| **Problems related to care-provider dependency** | Z74 | ICD-10 | 1.1 |
| **Other soft tissue disorders, not elsewhere classified** | M79 | ICD-10 | 1.1 |
| **Fall involving bed** | W06 | ICD-10 | 1.1 |
| **Open wound of head** | S01 | ICD-10 | 1.1 |
| **Other bacterial intestinal infections** | A04 | ICD-10 | 1.1 |
| **Diarrhoea and gastroenteritis of presumed infectious origin** | A09 | ICD-10 | 1.1 |
| **Pneumonia, organism unspecified** | J18 | ICD-10 | 1.1 |
| **Pneumonitis due to solids and liquids** | J69 | ICD-10 | 1 |
| **Speech disturbances, not elsewhere classified** | R47 | ICD-10 | 1 |
| **Vitamin D deficiency** | E55 | ICD-10 | 1 |
| **Artificial opening status** | Z93 | ICD-10 | 1 |
| **Gangrene, not elsewhere classified** | R02 | ICD-10 | 1 |
| **Symptoms and signs concerning food and fluid intake** | R63 | ICD-10 | 0.9 |
| **Other hearing loss** | H91 | ICD-10 | 0.9 |
| **Fall on and from stairs and steps** | W10 | ICD-10 | 0.9 |
| **Fall on same level from slipping, tripping and stumbling** | W01 | ICD-10 | 0.9 |
| **Thyrotoxicosis [hyperthyroidism]** | E05 | ICD-10 | 0.9 |
| **Scoliosis** | M41 | ICD-10 | 0.9 |
| **Dysphagia** | R13 | ICD-10 | 0.8 |
| **Dependence on enabling machines and devices** | Z99 | ICD-10 | 0.8 |
| **Agent resistant to penicillin and related antibiotics** | U80 | ICD-10 | 0.8 |
| **Osteoporosis with pathological fracture** | M80 | ICD-10 | 0.8 |
| **Other diseases of digestive system** | K92 | ICD-10 | 0.8 |
| **Cerebral Infarction** | I63 | ICD-10 | 0.8 |
| **Calculus of kidney and ureter** | N20 | ICD-10 | 0.7 |
| **Mental and behavioural disorders due to use of alcohol** | F10 | ICD-10 | 0.7 |
| **Other medical procedures as the cause of abnormal reaction of the patient** | Y84 | ICD-10 | 0.7 |
| **Abnormalities of heartbeat** | R00 | ICD-10 | 0.7 |
| **Unspecified acute lower respiratory infection** | J22 | ICD-10 | 0.7 |
| **Problems related to life-management difficulty** | Z73 | ICD-10 | 0.6 |
| **Other abnormal findings of blood chemistry** | R79 | ICD-10 | 0.6 |
| **Personal history of risk-factors, not elsewhere classified** | Z91 | ICD-10 | 0.5 |
| **Open wound of forearm** | S51 | ICD-10 | 0.5 |
| **Depressive episode** | F32 | ICD-10 | 0.5 |
| **Spinal stenosis (secondary code only)** | M48 | ICD-10 | 0.5 |
| **Disorders of mineral metabolism** | E83 | ICD-10 | 0.4 |
| **Polyarthrosis** | M15 | ICD-10 | 0.4 |
| **Other anaemias** | D64 | ICD-10 | 0.4 |
| **Other local infections of skin and subcutaneous tissue** | L08 | ICD-10 | 0.4 |
| **Nausea and vomiting** | R11 | ICD-10 | 0.3 |
| **Other noninfective gastroenteritis and colitis** | K52 | ICD-10 | 0.3 |
| **Fever of unknown origin** | R50 | ICD-10 | 0.1 |

**Abbreviations:** ICD-10 – International Classification of Diseases 10^th^ edition.

**Appendix 2. STROBE (Strengthening The Reporting of OBservational Studies in Epidemiology) Checklist**

| **Section/Topic** | Item No. | Recommendation | Reported on Page No. |
| --- | --- | --- | --- |
| **Title and abstract** | 1 | (*a*) Indicate the study’s design with a commonly used term in the title or the abstract | 2 |
|  |  | (*b*) Provide in the abstract an informative and balanced summary of what was done and what was found | 2 |
| Introduction | | | |
| Background/rationale | 2 | Explain the scientific background and rationale for the investigation being reported | 4 |
| Objectives | 3 | State specific objectives, including any prespecified hypotheses | 4 |
| Methods | | | |
| Study design | 4 | Present key elements of study design early in the paper | 5 |
| Setting | 5 | Describe the setting, locations, and relevant dates, including periods of recruitment, exposure, follow-up, and data collection | 5 |
| Participants | 6 | (*a*) *Cohort study*—Give the eligibility criteria, and the sources and methods of selection of participants. Describe methods of follow-up  *Case-control study*—Give the eligibility criteria, and the sources and methods of case ascertainment and control selection. Give the rationale for the choice of cases and controls  *Cross-sectional study*—Give the eligibility criteria, and the sources and methods of selection of participants | 5  /  / |
|  |  | (*b*) *Cohort study*—For matched studies, give matching criteria and number of exposed and unexposed  *Case-control study*—For matched studies, give matching criteria and the number of controls per case | /  / |
| Variables | 7 | Clearly define all outcomes, exposures, predictors, potential confounders, and effect modifiers. Give diagnostic criteria, if applicable | 5 |
| Data sources/measurement | 8* | For each variable of interest, give sources of data and details of methods of assessment (measurement). Describe comparability of assessment methods if there is more than one group | 5 |
| Bias | 9 | Describe any efforts to address potential sources of bias | / |
| Study size | 10 | Explain how the study size was arrived at | 5-6 |
| Quantitative variables | 11 | Explain how quantitative variables were handled in the analyses. If applicable, describe which groupings were chosen and why | 5-6 |
| Statistical methods | 12 | (*a*) Describe all statistical methods, including those used to control for confounding | 5 |
|  |  | (*b*) Describe any methods used to examine subgroups and interactions | 5 |
|  |  | (*c*) Explain how missing data were addressed | 5 |
|  |  | (*d*) *Cohort study*—If applicable, explain how loss to follow-up was addressed  *Case-control study*—If applicable, explain how matching of cases and controls was addressed  *Cross-sectional study*—If applicable, describe analytical methods taking account of sampling strategy | /  /  / |
|  |  | (*e*) Describe any sensitivity analyses | / |
| **Results** |  |  |  |
| Participants | 13* | (a) Report numbers of individuals at each stage of study—eg numbers potentially eligible, examined for eligibility, confirmed eligible, included in the study, completing follow-up, and analysed | / |
|  |  | (b) Give reasons for non-participation at each stage | / |
|  |  | (c) Consider use of a flow diagram | Figure S1 |
| Descriptive data | 14* | (a) Give characteristics of study participants (eg demographic, clinical, social) and information on exposures and potential confounders | 7-8 |
|  |  | (b) Indicate number of participants with missing data for each variable of interest | Figure S1 |
|  |  | (c) *Cohort study*—Summarise follow-up time (eg, average and total amount) | / |
| Outcome data | 15* | *Cohort study*—Report numbers of outcome events or summary measures over time | 7-9 |
|  |  | *Case-control study—*Report numbers in each exposure category, or summary measures of exposure | / |
|  |  | *Cross-sectional study—*Report numbers of outcome events or summary measures | / |
| Main results | 16 | (*a*) Give unadjusted estimates and, if applicable, confounder-adjusted estimates and their precision (eg, 95% confidence interval). Make clear which confounders were adjusted for and why they were included | 7-9 |
|  |  | (*b*) Report category boundaries when continuous variables were categorized | / |
|  |  | (*c*) If relevant, consider translating estimates of relative risk into absolute risk for a meaningful time period | / |
| Other analyses | 17 | Report other analyses done—eg analyses of subgroups and interactions, and sensitivity analyses | 7-9 |
| **Discussion** |  |  |  |
| Key results | 18 | Summarise key results with reference to study objectives | 10 |
| Limitations | 19 | Discuss limitations of the study, taking into account sources of potential bias or imprecision. Discuss both direction and magnitude of any potential bias | 14 |
| Interpretation | 20 | Give a cautious overall interpretation of results considering objectives, limitations, multiplicity of analyses, results from similar studies, and other relevant evidence | 10-14 |
| Generalisability | 21 | Discuss the generalisability (external validity) of the study results | 10-14 |
| **Other Information** |  |  |  |
| Funding | 22 | Give the source of funding and the role of the funders for the present study and, if applicable, for the original study on which the present article is based | 15 |
